# Complex-mediated evasion: modeling defense against antimicrobial peptides with application to human-pathogenic fungus *Candida albicans*

**DOI:** 10.1038/s41540-025-00559-1

**Published:** 2025-07-22

**Authors:** Yann Bachelot, Anastasia Solomatina, Marc Thilo Figge

**Affiliations:** 1 Applied Systems Biology, Leibniz Institute for Natural Product Research and Infection Biology, Hans Knöll Institute (HKI), Jena, Germany; 2 Faculty of Biological Sciences, Friedrich Schiller University, Jena, Germany; 3 Institute of Microbiology, Faculty of Biological Sciences, Friedrich Schiller University, Jena, Germany

**Keywords:** Computer modelling, Immunology

## Abstract

Understanding the complex interplay between host and pathogen during infection is critical for developing diagnostics and improving therapeutic interventions. Among the diverse arsenal employed by the host, antimicrobial peptides (AMP) play a key role in the defense against pathogens. We propose an immune evasion mechanism termed “Complex-mediated evasion” (CME), that allows pathogens to protect themselves against AMP and investigate it through mathematical modeling and computer simulations. To achieve CME, we hypothesize that the pathogen secretes defense molecules that bind AMP. When bound within the complex, AMP are unable to harm the pathogen. Due to molecular gradients, complexes may diffuse away from the pathogen, enhancing the protective effect of the mechanism by decreasing the concentration of AMP in the vicinity of the pathogen. We establish a mathematical model to (i) explore the sensitivity of the mechanism to various parameters and (ii) simulate the immune evasion of the human-pathogenic fungus *Candida albicans*.

## Introduction

Understanding the mechanisms of interaction between a pathogen and its host is of primary interest for infection prevention and the development of treatment strategies. Host-pathogen interactions occur across various spatial and temporal scales, involving molecular interactions, cell-cell, and cell-organ communication. In this study, we will focus on the innate immune response against pathogens during the early stage of an infection, where molecular interactions play an important role between host cells and pathogen cells^1^.

The release of antimicrobial peptides (AMP) by various cells, including immune cells such as neutrophils and epithelial cells^2,3^, represents an important defense mechanism activated by the innate immune system to clear infections. These small molecules, consisting of 10–60 amino acids^4^, are secreted in the extracellular space and have anti-microbial properties. Their mode of action relies on targeting the cell membrane of pathogens as well as their intracellular components. For instance, the human AMP LL-37 from the cathelicidin family disrupts the membrane of pathogen cells ultimately causing their death. Previous studies showed that for sufficiently high concentrations, LL-37 has lethal effects on some pathogens such as the yeast *Candida albicans*^5^. This makes AMP promising candidates for therapeutic interventions^6–8^.

*C. albicans* is a human commensal and opportunistic pathogen. Naturally occurring on the skin and mucosal surfaces, it can become human-pathogenic under immunosuppressive conditions, which is particularly dangerous for immunocompromised individuals. When becoming invasive, hyphae of *C. albicans* cells reach the bloodstream, and yeast cells may distribute throughout the whole body within a few hours, resulting in high mortality rates up to 60%^9^. In fact, the pathogen was listed in the critical priority group for human-pathogenic fungi according to the World Health Organization^10^. Despite the remarkable defense capacity of the immune system, pathogens have evolved sophisticated strategies to counteract host defenses^11^. This is also the case for *C. albicans*, which employs various mechanisms to avoid the immune response. For example, studies of bloodstream infections based on the whole-blood infection assay revealed that *Candida* cells can successfully evade the innate immune response in humans^12,13^ as well as in avian species^14^. While it is known that *C. albicans* can alter its morphology by growing hyphae, which protect it from phagocytosis by immune cells^15,16^, this cannot be its only strategy in immune evasion, as no substantial hyphal growth was observed in whole-blood assays. Interestingly, in the assays, some pathogen cells not only avoid phagocytosis but also mostly remain alive^17,18^. This suggests that certain pathogens, such as *C. albicans* and *C. glabrata*, can counteract AMP attacks without growing hyphae.

Experimental studies suggest that *C. albicans* uses a three-fold strategy to evade AMP attack. This includes the secretion of proteases that can degrade human AMP, such as sap9 and sap10, known to cleave and inactivate the histatin-5^19^. *C. albicans* also uses efflux pumps like Cdr1 and Cdr2 to expel AMP from its cytoplasm^20^. Finally, it was shown that *C. albicans* can secrete a glycoprotein fragment of the surface protein Msb2 into extracellular space that can bind to AMP, which offers the pathogen cell long-range protection against AMP^21,22^. It was shown that the glycoprotein Msb2* protects the pathogen cell from the effect of different human AMP, such as LL-37 and histatin-5, by binding to these AMP.

**Fig. 1:**
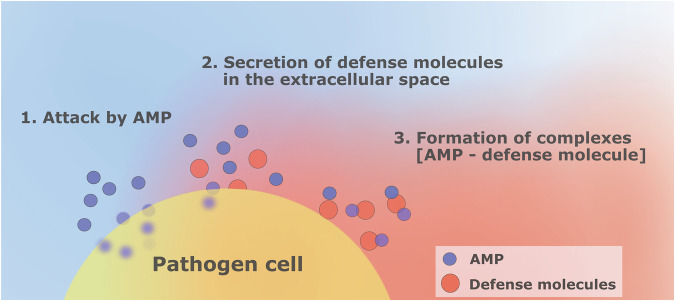
Complex-mediated evasion mechanism. The pathogen cell (yellow) is surrounded by AMP (blue) and secretes defense molecules (red). These molecules bind to AMP forming complexes in the extracellular space, preventing AMP from harming the pathogen cell.

The present study focuses on the complex-mediated evasion (CME) mechanism depicted in Fig. 1. To protect itself from the toxicity of AMP (item 1 in Fig. 1), the pathogen cell secretes defense molecules that diffuse in the extracellular space and bind AMP (items 2 & 3 in Fig. 1). When in the complex with the secreted molecules [AMP—defense molecule], AMP cannot harm the pathogen cell. Besides that, due to the concentration gradient of the molecules, the complexes may diffuse away from the pathogen cell, leading to a further decrease of AMP concentration in the vicinity of the pathogen. As a result, the pathogen evades the action of the immune system and increases its chance of survival.

Designing experiments to measure spatiotemporal concentrations of molecules is crucial to providing a better understanding of the proposed immune evasion mechanism, but represents a major challenge. In a systems biology approach, computational modeling helps bridge these experimental gaps enabling the exploration, analysis, and prediction of spatiotemporal dynamics of molecular interactions. This approach has been used in the past and applied to study the dynamics of the innate immune response against fungal pathogens in various infection scenarios using a variety of mathematical modeling approaches. Individual-based models, such as state-based models and agent-based models (ABM), were used to study fungal infection in blood^12,13,18^ as well as treatment strategies for neutropenic patients^23^ and assessment of immune cell function in blood from patients undergoing cardiac surgery^24^. Ordinary and partial differential equations (ODEs/PDEs) were used to model the self vs. non-self discrimination in *C. albicans* infection^25^ as well as *C. albicans* treatment of vulvovaginal infections using nanobodies^26^. Hybrid ABM, which combine ABM and ODEs/PDEs, have also been used to describe the early dynamics of the immune response against *Aspergillus fumigatus* infection in the lung of humans^27–32^ and mice^33,34^. In the present study, we model the CME mechanism using PDEs, which allows us to simulate spatiotemporal molecular dynamics at the population level. While not offering the individual-based resolution of an ABM, the lower computational cost of PDEs allows for extensive parameter screenings and sensitivity analyses. This model provides new insight into a critical but understudied defense strategy and allows us to investigate mechanistically how pathogen can evade AMP.

We first present the theoretical basis of CME by formulating PDEs, exploring the parameter space, and analyzing their effect on the model predictions. This enables gaining general insight into the immune evasion mechanism as well as identifying the necessary conditions for the pathogen to achieve immune evasion. Next, we study CME in the case of *C. albicans* infection. By combining the model results with experimental studies, we simulate *C. albicans* AMP evasion under different conditions as identified from parameter screenings.

## Results

**Fig. 2:**
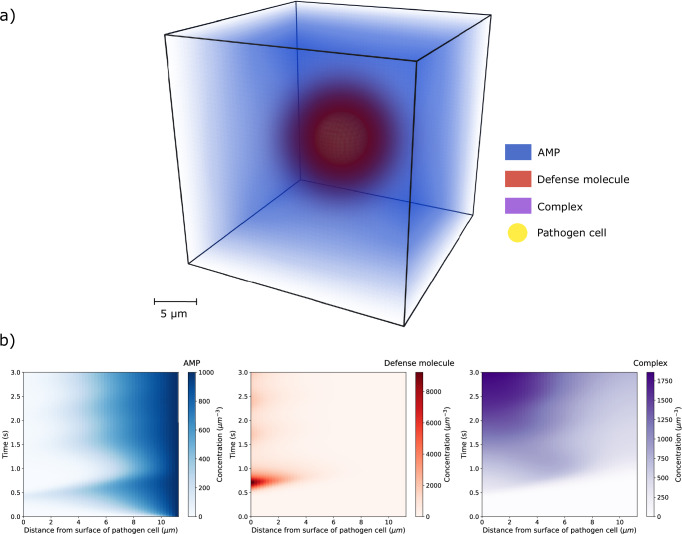
Visual representations of an example simulation for dynCME. The parameters used for the dynCME example simulation are presented in Supplementary Table 2. **a** Visual representation of the system as a simulation snapshot at $t=0.75\,{\rm{s}}$. The system is defined as a three-dimensional cube, with the pathogen cell modeled as a sphere centered in the environment. AMP (blue), defensive (red), and complexes (purple) molecules diffuse in the extracellular space and interact with each other as well as with the pathogen cell (yellow). **b** Spatiotemporal distributions of AMP, defense molecules, and complexes during $t=3.0\,{\rm{s}}$ represented as two-dimensional kymographs. The x-axis indicates the distance to the pathogen cell (spatial axis), while the y-axis corresponds to the time (temporal axis).

This study aims to investigate a pathogen’s immune evasion mechanism through computational modeling and theoretical analysis. First, we explore the CME mechanism as a general theoretical concept for the AMP evasion mechanism of an arbitrary pathogen cell. This is realized by introducing and comparing various models (see subsection “Model systems” in the “Methods” section), for which we performed extended screenings of the models’ parameters across different orders of magnitude (see Supplementary Table 1). This enables the characterization of the model’s sensitivity to specific parameters and the identification of parameter regimes where CME becomes effective. Two versions of CME are investigated in this study, which differ in the way how AMP availability is modeled. In the constant CME (conCME), we model a one-time treatment starting from a constant AMP distribution in space, whereas in the dynamic CME (dynCME) we implicitly model a dynamic process of AMP secretion by immune cells. Visual representations of the conCME (Supplementary Movie 1) and dynCME (Fig. 2a and Supplementary Movie 2) demonstrate the spatiotemporal dynamics of the mechanism for an exemplary parameter set during 2.0 s of simulated time. As shown in Fig. 2b, AMP flow into the system from the environmental surfaces and eventually reach the pathogen cell. Then, after a certain delay, the secretion of defense molecules starts on the pathogen cell surface. As a result, defense molecules bind the AMP, effectively decreasing its concentration. For the exemplary parameter set, this leads to a reduction of the AMP concentration close to the pathogen cell.

Second, we present an application of CME with the human-pathogenic fungus *C. albicans*. By combining modeling with experimental data^21,22^, we further characterize the immune evasion of the human-pathogenic fungus.

### conCME: simulations predict robust protection against one-time AMP treatment

**Fig. 3:**
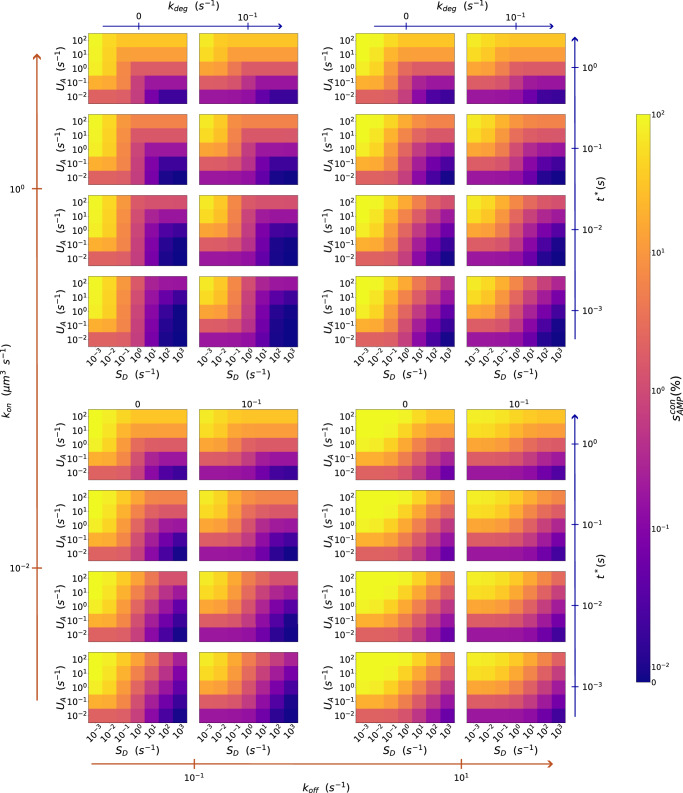
Reaction rate parameter screening of conCME model. Screening over six reaction rate parameters as presented in Supplementary Table 1. The color bar shows the ${s}_{\mathrm{AMP}}^{\mathrm{con}}$ score on a log scale. For example, the orange color corresponds to a score of 10%, indicating that 10% of the initial concentration of AMP has been taken up by the pathogen.

In this scenario, the initial state of the system is characterized by AMP homogeneously distributed around the pathogen cell (see subsection “conCME: Model with constant AMP concentration” in the “Methods” section). This scenario can be interpreted as a one-time medical treatment, mimicking one dose of AMP added to the system. As summarized in Supplementary Table 1, for this model, we performed a screening of the reaction rate parameters over several orders of magnitude. The diffusion coefficients were fixed to $40\,{\mathrm{\mu m}}^{2}\,{{\rm{s}}}^{-1}$ for AMP and defense molecules and $20\,{\mathrm{\mu m}}^{2}\,{{\rm{s}}}^{-1}$ for the complex. For each parameter combination, the simulation was stopped when the AMP concentration reached the steady state, which in this scenario corresponds to the extinction of extracellular AMP. To estimate the protective effect of CME, we introduced the ${s}_{\mathrm{AMP}}^{\mathrm{con}}$ score (see subsection “AMP score for conCME simulations” in the “Methods” section), which corresponds to the fraction of the AMP concentration taken up by the pathogen, ${s}_{{\rm{A}}{\rm{M}}{\rm{P}}}^{{\rm{c}}{\rm{o}}{\rm{n}}}=\frac{{[A]}_{{\rm{t}}{\rm{a}}{\rm{k}}{\rm{e}}{\rm{n}}{\rm{u}}{\rm{p}}}^{t=\infty }}{{[A]}^{t=0}}\times 100$, and plotted it in a heatmap representation shown in Fig. 3. Each square on the heatmap corresponds to a unique parameter set, and the color represents the ${s}_{\mathrm{AMP}}^{\mathrm{con}}$ score. The parameter combination used for a particular simulation can be read from the six different axes: the two outmost axes (orange) correspond to the ${k}_{\mathrm{on}}$ and ${k}_{\mathrm{off}}$ parameters, the middle axes (blue) represent *k*_deg_ and ${t}^{* }$, and finally ${U}_{A}$ and ${S}_{D}$ are shown on the two inner axes. Figure 3 shows part of the screening data, while the complete results of the screening can be found in Supplementary Fig. 1.

Parameter screening of the conCME model reveals parameter regimes with high ${s}_{\mathrm{AMP}}^{\mathrm{con}}$ scores, corresponding to AMP efficiently damaging the pathogen cell, represented in yellow in Fig. 3. For AMP to harm the pathogen, they need to have a high uptake rate parameter by the pathogen (${U}_{A}\ge 10\,{{\rm{s}}}^{-1}$, two top rows of each subplot). At the same time, the defense molecules are more efficient (boxes shown with dark blue in Fig. 3 and corresponding to low ${s}_{\mathrm{AMP}}^{\mathrm{con}}$ scores) if they are secreted in high amounts or if they have a high affinity with AMP. For example, for simulations with the parameter related to the secretion rate ${S}_{D}\ge 1.0\,{{\rm{s}}}^{-1}$, at most 10% of the initial concentration of AMP is taken up. In addition, a short reaction time ${t}^{* }$ of the pathogen also results in lower ${s}_{\mathrm{AMP}}^{\mathrm{con}}$ scores, indicating the regime beneficial for the pathogen cell.

The results of the screening showcased a reduction in the AMP uptake across all the parameter space explored. From all the parameter combinations, 88% of the simulations resulted in a reduction of at least 50% in the AMP uptake (${s}_{\mathrm{AMP}}^{\mathrm{con}} < 50 \%$), when compared to the case with no defense. Moreover, 65% of the simulations yielded a score ${s}_{\mathrm{AMP}}^{\mathrm{con}} < 10 \%$, implying that CME is a robust mechanism for pathogens to evade the harmful effect of AMP.

### conCME: global sensitivity analysis reveals reactions on pathogen cell surface driving CME

**Fig. 4:**
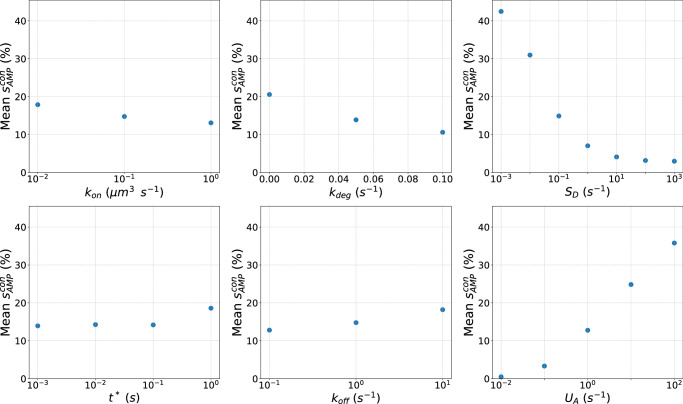
Parameters effect of conCME. Partial dependence plots. Each subplot corresponds to one reaction rate parameter, with the mean score ${s}_{\mathrm{AMP}}^{\mathrm{con}}$ (%) computed for each unique parameter value screened.

We investigated how each reaction rate parameter influenced the conCME simulation outcome, specifically focusing on the score ${s}_{\mathrm{AMP}}^{\mathrm{con}}$. To analyze the impact of individual parameters, we separated the simulations by their unique values. We then computed the mean score ${s}_{\mathrm{AMP}}^{\mathrm{con}}$ within each cross-section of the parameter space, corresponding to these unique values of parameters. This allowed us to assess the overall effect of each parameter on the simulation results using partial dependence plots. The results, shown in Fig. 4, indicate that the three parameters ${k}_{\mathrm{on}}$, *k*_deg_ and ${S}_{D}$ tend to reduce the score ${s}_{{AMP}}^{{con}}$, meaning that higher values of these parameters result in less AMP uptake by the pathogen cell (first row in Fig. 4). Higher values of the other three parameters ${t}^{* }$, ${k}_{\mathrm{off}}$ and ${U}_{A}$ result in higher mean scores ${s}_{\mathrm{AMP}}^{\mathrm{con}}$ (second row in Fig. 4). The uptake rate parameter ${U}_{A}$ and secretion-related rate parameter ${S}_{D}$ have the largest impact on the simulation outcome, as shown by the range in the mean score ${s}_{\mathrm{AMP}}^{\mathrm{con}}$ in the partial dependence plots. This observation is expected given that ${U}_{A}$ directly reflects the efficiency with which AMP are taken up by the pathogen cell once they have reached the cell membrane, while ${S}_{D}$ describes how many defense molecules are secreted by the pathogen. The sensitivity analysis results were further supported by the SHAP analysis performed on a surrogate model that first was trained and validated to predict the score ${s}_{\mathrm{AMP}}^{\mathrm{con}}$ based on the conCME reaction rate parameters (see Supplementary Note 1).

Overall, the results of the SHAP analysis of the surrogate model (Supplementary Fig. 2b) confirmed what was observed in Fig. 4. The uptake rate parameter ${U}_{A}$ and the parameter related to the secretion rate ${S}_{D}$ drive the outcome of the simulation; both parameters are involved in reactions happening at the pathogen cell membrane. The more AMP are taken up (high ${U}_{A}$), the higher is ${s}_{\mathrm{AMP}}^{\mathrm{con}}$. To defend against AMP, the pathogen cell needs to secrete defense molecules in the largest possible quantities (high ${S}_{D}$) and as early as possible (low ${t}^{* }$). The effect of the diffusion constants of the different molecules was also investigated (see Supplementary Note 2 and Supplementary Fig. 3). Within the screened range, the diffusion coefficients only had a minor effect on the outcome of the simulations.

### conCME: diffusion of molecular complexes defines two regimes of system behavior

One of the properties of CME mechanism is that AMP are transported away from the pathogen cell through the formation of complexes due to their concentration gradient. Since defense molecules bind to AMP but do not degrade them, the diffusion of complexes away from the pathogen can enhance CME, by ensuring that in the event of complex dissociation, AMP are no longer harmful to the pathogen cell. To determine how this mechanism of diffusion of complexes [AMP—defense molecule] affects the CME, we performed reference simulations for the screened reaction rate parameters using the conCME with the diffusion of complexes being switched off. As a result, the formed complexes remain localized at their site of formation. Although these reference simulations are not realistic, by quantitative comparison to simulations with diffusion of complexes, its effect can be evaluated.

The comparison analysis was performed by computing the relative difference ($\mathrm{RD}$) in the ${s}_{\mathrm{AMP}}^{\mathrm{con}}$ score between the two scenarios for each of the parameter combinations from the parameter screening. Here, we computed ${s20}_{\mathrm{AMP}}^{\mathrm{con}}$ corresponding to initial simulations with ${D}_{C}=20\,{\mathrm{\mu m}}^{2}\ {{\rm{s}}}^{-1}$ and ${s0}_{\mathrm{AMP}}^{\mathrm{con}}$ corresponding to simulations with ${D}_{C}=0\,{\mathrm{\mu m}}^{2}\ {{\rm{s}}}^{-1}$: ${RD}=\frac{{s20}_{\mathrm{AMP}}^{\mathrm{con}}-{s0}_{\mathrm{AMP}}^{\mathrm{con}}}{{s0}_{\mathrm{AMP}}^{\mathrm{con}}}$. From this analysis, two regimes were found: the first regime with $\mathrm{RD} < 0$ corresponds to immune evasion being promoted by the passive transport of the complex [AMP—defense molecule] away from the pathogen cell. This regime was identified for 70% of the simulations within the explored parameter space. The second regime with $\mathrm{RD} > 0$ was found for parameter combinations, for which it is beneficial for the pathogen cell if the complexes are not diffusing, which was observed for the rest 30% of the simulations.

**Fig. 5:**
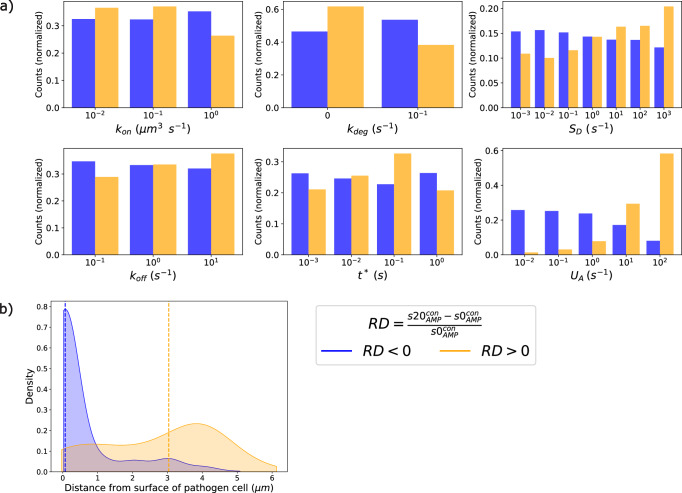
Comparison of two regimes: Diffusion of complexes promoting CME ($\mathrm{RD} < 0$, blue) or inhibiting CME ($\mathrm{RD} > 0$, orange). **a** Distribution of the parameter values in both regimes. **b** Spatial distribution of the formation of complexes. The dotted lines represent the median value.

To further characterize the conditions needed to achieve each of the regimes, the distributions of the unique parameter values per regime were computed and gathered in Fig. 5a. The plot presents the fraction of each parameter value relative to the number of simulations per regime. The regime corresponding to $\mathrm{RD} > 0$ (orange) was mainly found for a high uptake rate parameter ${U}_{A}$ and was more frequently observed for a high secretion-related rate parameter ${S}_{D}$, as shown by the subplots for ${U}_{A}$ and ${S}_{D}$ in Fig. 5a. We also observed that the $\mathrm{RD} < 0$ regime (blue) was slightly more frequently obtained for high degradation rates (see subplot for *k*_deg_), which is due to AMP that are transported away from the pathogen cell, degrading more quickly than the time required to return to the pathogen cell.

Next, we aimed to better understand the dynamics in the two regimes, specifically investigating why the diffusion of complex enhances or impairs the pathogen’s immune evasion. We computed the spatial distributions of the formation of complexes throughout the whole simulation as a function of the distance from the surface of the pathogen cell. Figure 5b shows these spatial distributions for both regimes, which were obtained by retrieving the spatial position with the highest concentration of complexes formed during the simulations and collected in the density plot. In the $\mathrm{RD} < 0$ regime (blue), complexes formed predominantly close to the pathogen cell surface, making, therefore, the diffusion of complexes beneficial for the pathogen. This can also be seen in Supplementary Movies 3 and 4, which show the concentrations of complexes over time for an exemplary parameter combination, either in the presence (Supplementary Movie 3) or in the absence (Supplementary Movie 4) of diffusion of complexes. In contrast, in the $\mathrm{RD} > 0$ regime (orange), complexes formed far away from the pathogen. This can be seen in Supplementary Movies 5 and 6, which, similarly, show the concentration of complexes over time for an exemplary parameter set, either in the presence (Supplementary Movie 5) or in the absence (Supplementary Movie 6) of diffusion of complexes. Instead of transporting AMP away from the cell within the complexes, AMP were transported back towards the pathogen cell, resulting in higher ${s}_{\mathrm{AMP}}^{\mathrm{con}}$ scores. This was only possible if defense molecules were produced in excess by the pathogen.

These analyses demonstrate that diffusion of complexes enhances the protection of the pathogen cell only when the concentration of defense molecules is low (corresponds to a small secretion rate value ${S}_{D} < 1\,{\upmu {\rm{m}}}\ {{\rm{s}}}^{-1}$). In this case, if reactions occur faster than diffusion (Supplementary Note 3 and Supplementary Fig. 4), complexes form close to the pathogen surface and therefore their diffusion is beneficial for the pathogen cell. However, if diffusion occurs faster than reactions, complexes form far from the pathogen cell, with their diffusion resulting in a disadvantage for the pathogen cell. In scenarios where defense molecules are secreted in higher amounts (${S}_{D} > {1}\,{{\upmu {\rm{m}}}}\ {{\rm{s}}}^{-1}$), the diffusion of complexes is prejudicial for the pathogen cell. That is because defense molecules produced in excess diffuse and complexes form predominantly far away from the surface of the pathogen cell.

### dynCME: CME protects pathogen from AMP inflow

**Fig. 6:**
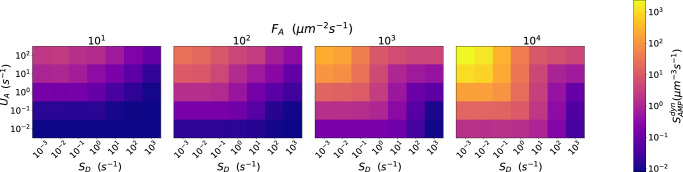
Parameter screening for dynCME. Screening over the three parameters ${{U}_{A},\,{S}_{D},{F}}_{A}$. The color bar shows the score ${s}_{\mathrm{AMP}}^{\mathrm{dyn}}$ on a log scale. Low values correspond to the regime of immune evasion and are shown in dark blue, whereas high values are shown in yellow. The results presented here correspond to the parameter subspace ${k}_{{\rm{o}}{\rm{n}}}={10}^{-2}{\mathrm{\mu m}}^{3}\,\cdot \,{{\rm{s}}}^{-1},\,{k}_{{\rm{o}}{\rm{f}}{\rm{f}}}={10}^{-1}\,{{\rm{s}}}^{-1},\,{k}_{{\rm{d}}{\rm{e}}{\rm{g}}}=0\,{{\rm{s}}}^{-1}$ and ${t}^{* }={10}^{-3}{\rm{s}}$.

Performing dynCME simulations, we modeled the dynamics of AMP inflow at the boundaries of the cubic simulation space to mimic AMP secretion by immune cells in the distant environment (see subsection “dynCME: Model for AMP inflow” in the “Methods” section). We introduced the score ${s}_{\mathrm{AMP}}^{\mathrm{dyn}}$, which is an AMP uptake per time unit, i.e., ${s}_{\mathrm{AMP}}^{\mathrm{dyn}}=\frac{{[A]}_{\mathrm{takenup}}^{t=\infty }}{\triangle t}$, where $\triangle t$ is the timestep used in the simulation, and computed it for each parameter set at the non-equilibrium steady state. Figure 6 shows the result of the parameter screening of the parameter subspace corresponding to the fixed values ${k}_{\mathrm{on}}={10}^{-2}\,{{\upmu m}}^{3}\ {{\rm{s}}}^{-1},\,{k}_{\mathrm{off}}={10}^{-1}\,{{\rm{s}}}^{-1}$ and ${t}^{* }={10}^{-3\,}{\rm{s}}$, while the complete parameter screening is given in Supplementary Fig. 5. Dark blue boxes represent simulations with ${s}_{\mathrm{AMP}}^{\mathrm{dyn}}\,\le \,{10}^{-1}\,{{\upmu m}}^{-3}{\ {\rm{s}}}^{-1}$ corresponding to the scenario, when the pathogen is able to evade most of the AMP attack by their neutralization before they reach the pathogen surface, with only a relatively small fraction of AMP uptake by the pathogen. This was observed for 32% of all the simulations across the parameter space. In contrast, simulations with ${s}_{\mathrm{AMP}}^{\mathrm{dyn}}\,\ge \,{10}^{2}\,{\mathrm{\mu m}}^{-3}{\ {\rm{s}}}^{-1}$, indicated by orange and yellow boxes in Fig. 6, demonstrated that the pathogen cell was unable to neutralize AMP by secretion of defense molecules. This regime was found for 8% of the simulations. Figure 6 demonstrates that the higher the inflow of AMP, denoted by the flux parameter ${F}_{A}$, the more AMP were taken up by the pathogen cell and the higher was the score ${s}_{\mathrm{AMP}}^{\mathrm{dyn}}$. The most efficient way for the host to harm the pathogen was for the combination of high inflow flux parameter ${F}_{A}$ and its high uptake rate at the surface of the pathogen cell ${U}_{A}.$ For high concentrations of AMP coming towards the pathogen cell (${F}_{A}\ge {10}^{3}\,{{\upmu m}}^{-2}\ {{\rm{s}}}^{-1}$), the pathogen needed to produce and secrete defense molecules in high concentrations $({S}_{D}\ge 10\,{{\rm{s}}}^{-1})$ to protect itself. As shown in Supplementary Fig. 5, the parameter ${t}^{* }$, which corresponds to the time needed by the pathogen to start secreting defense molecules after initial AMP sensing, had little to no effect on the readout. This is due to the score ${s}_{\mathrm{AMP}}^{\mathrm{dyn}}$ depending only on the non-equilibrium steady state and not on the cumulative AMP that is taken up during the whole simulation time. The two other parameters related to the binding kinetics (${k}_{\mathrm{on}}$ and ${k}_{\mathrm{off}}$) had similar effects as for the conCME simulations; that is the pathogen benefited from a lower dissociation constant $\frac{{k}_{\mathrm{off}}}{{k}_{\mathrm{on}}}$ between secreted defense molecules and AMP.

The addressed scenario involving AMP inflow on the boundaries of the simulated spatial domain sheds light on long-term interactions between the pathogen and the host. We demonstrated that the proposed immune evasion mechanism allows the pathogen cell to not only be able to defend itself against one wave of AMP, as in the one-treatment scenario modeled by conCME, but also when confronted with a hostile environment for a longer time as modeled by dynCME.

### Computer simulations reveal *C. albicans* LL-37 dependent survival probability based on experimental data

**Fig. 7:**
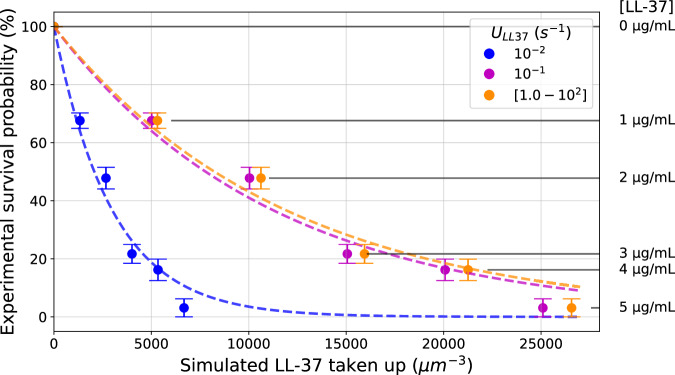
Relation between the simulated concentration of LL-37 taken up by the pathogen and its experimentally measured probability of survival. The x-axis represents the simulated concentration of LL-37 that was taken up during 1.5 h using conCME. The y-axis represents the probability of survival, which was measured experimentally in the survival assays performed in ref. ^21^, shown with standard deviation. The colors refer to the screened values of the uptake rate used in the simulations. The different experimental conditions, corresponding to the initial concentration of LL-37 in the system, are indicated on the right side of the plot. The dotted lines represent the exponential decay fitted (see Supplementary Table 5) to the experimental and simulated data that allow us to relate the concentration of LL-37 that was taken up by the pathogen cell to its probability of survival.

To model the AMP evasion of *C. albicans* based on existing experimental data^21^, we first established the relation between the probability of survival of the pathogen, as measured by colony forming units (CFU) in survival assays, and the concentration of LL-37 uptake, resulting from simulations using conCME. We used the experimental data of the survival assay for the *C. albicans* msb2Δsho1 mutant, which is a *Candida* strain that does not produce any Msb2* molecules^21^. *Candida* cells were incubated together with LL-37 and CFU counts were measured after one and a half hours. The size of the three-dimensional cubic environment used in the simulation was computed based on the measured concentration of *C. albicans* cells, and the diffusion coefficient computed based on the size of the molecules (see Supplementary Note 4). The initial concentration of LL-37 was varied according to the experiment and we assumed no degradation of LL-37 during the experimental time of 1.5 h. Due to the absence of defense molecules in this condition, the other reaction rate parameters such as ${k}_{\mathrm{on}}$, ${k}_{\mathrm{off}}$, ${S}_{D}$, and ${t}^{* }$ are not involved in the model. We simulated the experimental system in silico by screening over the only remaining unknown parameter for the uptake ${U}_{\mathrm{LL}37}$. Then, for each unique value of ${U}_{\mathrm{LL}37}$, we related the concentration of LL-37 that was taken up, given by the simulation, to the probability of survival, given by the experiment. We fitted to the resulting data points an exponential decay function bound between zero and 100: $\mathrm{Probability}\,\mathrm{of}\,\mathrm{survival}\,({\rm{x}})=100 \cdot {e}^{(-\mathrm{kx})}$, where $x$ denotes the concentration of LL-37 uptake and $k > 0$ the decay constant to be fitted to the experimental and simulated data as a function of ${U}_{\mathrm{LL}37}$. The results of the fitting are shown in Fig. 7 with the fitted decay rates reported in Supplementary Table 5, providing the relation between the experimental measurement (probability of survival) and the readout of the model simulations (LL-37 taken up).

### Computer simulations suggest high binding valency of Msb2* for LL-37 molecules

It has been suggested that Msb2* might have multiple binding sites for AMP, based on survival assays that showed pathogen protection even at low concentrations of Msb2* compared to higher LL-37 concentrations^21^. Based on the experimental survival assays, we estimated the binding valency of Msb2* using an extension of conCME that considers a varying binding valency and is referred to as conCMEval (see subsection “conCMEval: Model for varying binding valency of defense molecules” in the “Methods” section). For this, we simulated the conCMEval model under experimental conditions^21^, where *C. albicans* msb2Δ0sho1 cells were incubated with $3\,{\upmu g}\,{\mathrm{mL}}^{-1}$ of LL-37 and $10\,{\upmu g}\,{\mathrm{mL}}^{-1}$ of Msb2*, and CFUs were measured after 1.5 h, which revealed that $90\pm 6 \%$ of the *C. albicans* cells did survive.

**Fig. 8:**
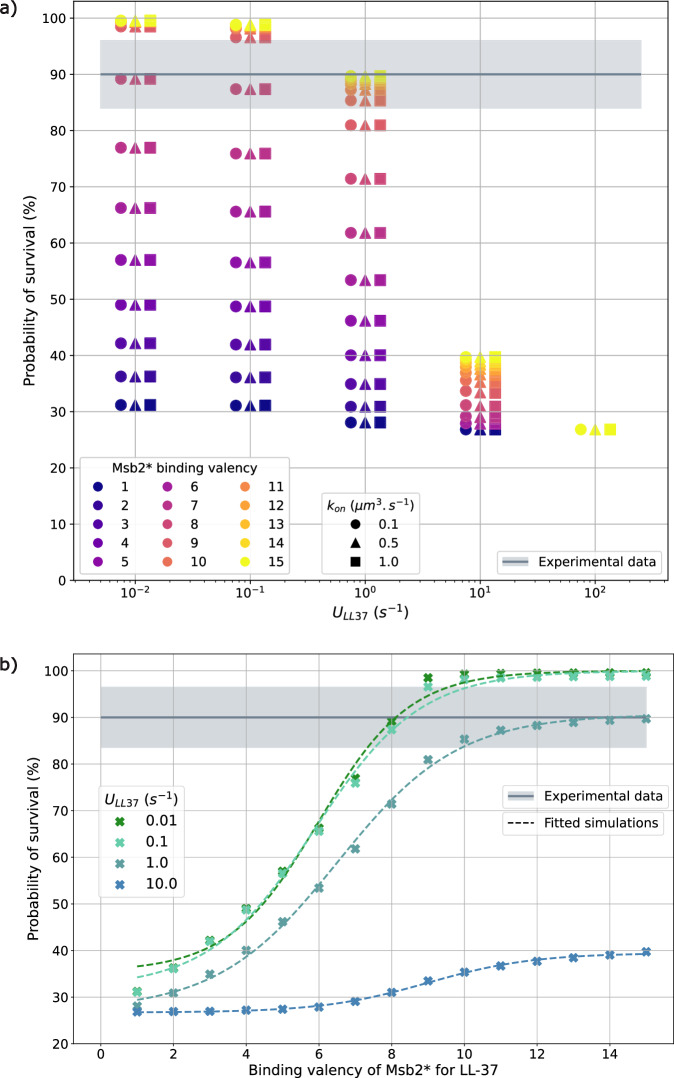
Simulation outcome for different numbers of LL-37 binding sites on Msb2*. **a**. The simulated survival probability of *C. albicans* is plotted as a function of three parameters: the uptake rate ${U}_{\mathrm{LL}37}$ (represented on x-axis), the number of binding sites used in simulation (indicated by different colors), and the association rate ${k}_{\mathrm{on}}$ (shown with markers’ shapes). The gray solid line and the shaded area, respectively, represent the mean and standard deviation of the experimental data^21^. **b**. The survival probability of the pathogen is plotted against the number of LL-37 binding sites on Msb2* for ${k}_{\mathrm{on}}={10}^{-1}\,{\mathrm{\mu m}}^{3}\,{{\rm{s}}}^{-1}$. The different colors refer to different values of the uptake rate ${U}_{\mathrm{LL}37}$.

Assuming that the experimental environment is well-mixed and, therefore, LL-37 and Msb2* are initially homogeneously distributed around the pathogen, we used conCMEval to screen for the three unknown parameters: uptake rate ${U}_{\mathrm{LL}37}$, association rate ${k}_{\mathrm{on}}$, and Msb2* binding valency. The dissociation rate ${k}_{\mathrm{off}}$ was computed for each ${k}_{\mathrm{on}}$ value based on dissociation constant ${k}_{D}$ measured experimentally in ref. ^22^. Parameter rates related to the secretion of Msb2* by the pathogen cell, such as ${S}_{\mathrm{Msb}2* }$ and ${t}^{* }$, are not considered here as the *C. albicans* mutant msb2Δ0sho1 do not produce Msb2*. Using the relation found between the concentration of AMP taken up and the pathogen survival probability given ${U}_{\mathrm{LL}37}$, we screened over the number of Msb2* binding sites varied from 1 to 15, and compared the simulated survival probabilities with the experimentally measured value ($90\,\pm \,6 \%$). Figure 8a shows the screening result and its comparison to the experimental probability of survival. As expected, a higher number of LL-37 binding sites on Msb2* increases the survival chances of *C. albicans* cells.

To match the experimental probability of survival in Fig. 8a, the screening suggested that a Msb2* molecule can bind at least eight LL-37 molecules, under the assumption that the binding rate ${k}_{\mathrm{on}}$ does not depend on the number of LL-37 molecules already bound by Msb2*. For fewer binding sites than eight, the pathogen does not have a sufficiently high probability of survival to be in line with the experimental results. Furthermore, we found that the association rate ${k}_{\mathrm{on}}$, shown in Fig. 8a with different markers for different values, does not affect the probability of survival of the pathogen cell, while the dissociation constant ${k}_{D}=\frac{{k}_{\mathrm{off}}}{{k}_{\mathrm{on}}}$ impacts the outcome. This can be confirmed by simplifying the conCMEval model to the mass-action kinetics for this particular experiment. Since both LL-37 and Msb2* molecules are homogeneously distributed, we can approximate this scenario to the following equilibrium: ${k}_{{on}}\,{[\mathrm{LL}37]}^{n}\left[\mathrm{Msb}{2}^{* }\right]=\,{k}_{\mathrm{off}}[C]$, where $n$ is the number of binding sites and $[C]$ is the concentration of the complexes. The concentration of complexes at the equilibrium depends only on the ratio $\frac{{k}_{\mathrm{on}}}{{k}_{\mathrm{off}}}$ (which was fixed according to ref. ^22^) and *n*. It is correlated with the total uptake of AMP as more complexes result in less free AMP that could harm the pathogen. Therefore, only the ratio ${k}_{D}=\frac{{k}_{\mathrm{off}}}{{k}_{\mathrm{on}}}$ impacts the outcome of the simulation in this specific scenario.

Figure 8b shows the relation between the probability of survival and the number of binding sites depending on the uptake rate ${U}_{\mathrm{LL}37}$. The fitting was achieved using a shifted logistic model: $f\left(x\right)=c+\frac{L}{1+{e}^{-k(x-x0)}}$, the fitted parameters are provided in Supplementary Table 6. The plot demonstrates that for numbers of binding sites larger than twelve, the probability of survival reaches a plateau and a higher valency does not offer more protection for the pathogen.

### Model predictions support AMP immune evasion of *C. albicans*

**Fig. 9:**
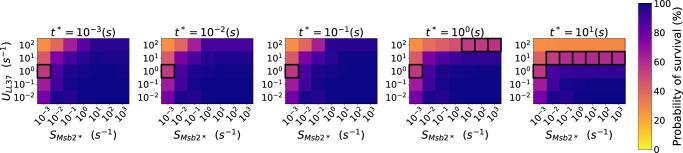
ConCMEval simulation outcome for different parameters for the pathogen *C. albicans.* The color bar shows the probability of survival of the pathogen for a particular parameter set. Higher values (dark blue) correspond to the immune-evasion regime, with high chances for the pathogen to survive, whereas lower values (orange) correspond to a probability of survival of around $30 \%$. Simulations that agree with experimental data (probability of survival between 50 and 60%) are highlighted with a black box. Parameter values used for the simulations are presented in Supplementary Table 4.

Using the estimated model parameters from experimental data with the conCMEval, we simulated a realistic infection scenario for *C. albicans*. The parameter screening results presented in Fig. 9 were obtained for the parameter values summarized in Supplementary Table 4. The simulations were carried out for 1.5 h real-time as in the survival assays, with LL-37 molecules initially distributed homogeneously around the *C. albicans* cell with concentration $3\,{\upmu g}\, \,{\mathrm{mL}}^{-1}$ as in experiments, where around 55% of the *C. albicans* cells did survive^22^. Figure 9 shows the simulation outcomes for the screening over the remaining unknown parameters, namely ${U}_{\mathrm{LL}37}$, ${t}^{* }$, and ${S}_{\mathrm{Msb}2* },$ where the latter denotes the secretion of Msb2* by *C. albicans*. The probability of survival of a pathogen cell is shown by the color bar in percentage.

Several parameter combinations led to a survival probability of the pathogen in accordance with the experimental results, as shown by the black borders of the colored boxes. This reinforces the hypothesis that the proposed CME mechanism is sufficient to explain the immune evasion of *C. albicans* against AMP, observed and quantified experimentally.

### CME is more efficient against low AMP inflow than against one-time treatment

Similar to the previous section, utilizing the estimated parameters based on the experimental data, we simulated CME in the realistic case of *C. albicans* in the presence of an inflow of AMP using the dynCMEval model (see subsection “dynCMEval: Model with AMP inflow for varying binding valency of defense molecules” in the “Methods” section). The total amount of AMP introduced in the system was adjusted for different flow rate parameter values ${F}_{\mathrm{LL}37}$ with corresponding times ${t}_{1}$, at which the inflow at the boundaries was switched off, such that the total amount of AMP was the same as for the one-time treatment scenario. This enables the comparison of CME for different conditions, such as for low and sustained inflow of AMP or for high inflow over a short period of time. All other parameters were screened as in the conCMEval simulations applied to *C. albicans*.

**Fig. 10:**
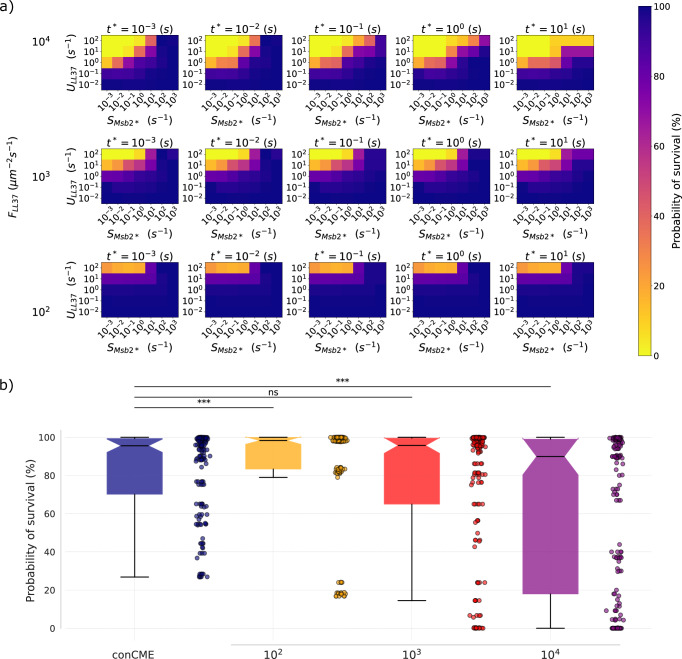
DynCMEval simulation outcome for different parameters for the pathogen *C. albicans* and its comparison to conCMEval simulations. **a** Survival probabilities of *C. albicans* for different parameter sets. The color shows the survival probability of the pathogen for a particular parameter set. Higher values (dark blue) correspond to the immune-evasion regime, with high chances for the pathogen to survive, whereas lower values (yellow) correspond to a probability of survival for the pathogen cell close to $0 \%$. Parameter values used in the simulations are presented in Supplementary Table 4. **b** Boxplot of the probability of survival of the parameter screening for the conCMEval simulations (blue), and for the different values of ${F}_{{LL}37}$ of the dynCMEval simulations. The black line on the boxes indicates the median value. The comparison between the two models was performed by a Kruskal-Wallis test. The significance shown by $* * *$ indicates ${P}_{\mathrm{val}} < 0.001$, and $\mathrm{ns}$ – ${P}_{val} > 0.05$.

The survival probabilities of the pathogen were computed for the different parameter combinations and are presented in Fig. 10a. High inflow of AMP for a short time resulted in lower survival probabilities for the pathogen than lower inflow values for a longer time, especially when the delay until secretion ${t}^{* }$ was high (${t}^{* }\ge {10}^{-1}\,{\rm{s}}$). For low values of inflow ${F}_{\mathrm{LL}37}={10}^{2}\,{\mathrm{\mu m}}^{-2}\, {{\rm{s}}}^{-1}$, ${t}^{* }$ had almost no effect on *C. albicans* survival. This can be explained by the low concentration of AMP in the system due to the low inflow of AMP at the system boundary, thus the uptake of LL-37 during the delay, before the secretion of Msb2* molecules, only represents a small fraction of the total uptake, such that its effect on the pathogen survival probability is negligible. Probabilities of survival of *C. albicans* below 50% were only found for a relatively high LL-37 uptake rates ${U}_{\mathrm{LL}37}\ge 10\,{{\rm{s}}}^{-1}$, combined with low secretion rate of Msb2* ${S}_{\mathrm{Msb}2* }$ and high inflow of LL-37 ${F}_{\mathrm{LL}37}$, showcasing the efficacy of CME in protecting the pathogen from AMP.

To further compare the one-time treatment scenario by AMP, simulated with the conCMEval, to the inflow of AMP simulated with the dynCMEval, the survival probability of *C. albicans* for the different parameter sets was represented in a boxplot in Fig. 10b. For the inflow flux parameter ${F}_{\mathrm{LL}37}={10}^{2}{\mathrm{\mu m}}^{-2}\,{{\rm{s}}}^{-1}$, the survival probabilities of *C. albicans* were significantly higher than in conCMEval simulations (${P}_{\mathrm{val}}\, < \,{10}^{-3}$). For ${F}_{\mathrm{LL}37}={10}^{3}\,{\mathrm{\mu m}}^{-2}\, {{\rm{s}}}^{-1}$, simulations resulted in comparable survival probabilities for the pathogen as in the conCMEval, as indicated by the non-significant differences based on statistical tests. This was expected as a high inflow of AMP for a very short time can be interpreted as the one-time treatment modeled by the conCMEval. Finally, for ${F}_{\mathrm{LL}37}={10}^{4}\,{\mathrm{\mu m}}^{-2}\,{{\rm{s}}}^{-1}$, the survival probabilities of *C. albicans* were significantly lower comparing to the conCMEval scenario (${P}_{\mathrm{val}}\, < \,{10}^{-3})$. These results suggest a more efficient protection of the pathogen against low and sustained inflow of AMP compared to one-time treatment or high inflow of AMP for a short period of time.

## Discussion

During infection, the host’s immune system and the invading pathogen interact, with each trying to assert itself. Both of them are equipped with various strategies to counteract the other. These interactions happen on diverse length and time scales comprising molecular and cellular levels. Understanding these interactions, especially regarding immune evasion mechanisms of pathogens, is of primary interest for the development of new treatments. Several studies^12,13^ focus on and model host-pathogen interactions at the cellular level, as experimental data is usually easier to obtain than at the molecular level. As a result, these models often make assumptions and simplifications about what happens on molecular scale, possibly missing on important processes.

This study investigated and modeled an AMP evasion mechanism, which we refer to as “Complex-mediated evasion” (CME), that may be employed by pathogens such as the human-pathogenic fungus *C. albicans*. CME is proposed to occur due to the secretion of defense molecules by the pathogen and the binding of these molecules to the AMP, leading to the effective decrease of AMP that can reach the pathogen’s surface. We formulated a PDE reaction-diffusion model that includes processes in the extracellular space (formation of the complex [AMP—defense molecule], its dissociation, and diffusion of all components), on the pathogen surface (AMP uptake, secretion of defense molecules), and on the system boundaries. The system was represented by a three-dimensional cubic environment with a single pathogen cell in its center. We used two different scenarios to introduce AMP into the system. The first one, referred to as conCME, corresponds to a homogeneous initial distribution of AMP around the pathogen cell and can be seen as a one-time AMP treatment when one dose of AMP is introduced to the system. This scenario was subsequently used to simulate in vitro experiments, where the initial condition is defined by a constant concentration of AMP. The second scenario, referred to as dynCME, mimicked a continuous production of AMP outside of the system, which was modeled by an inflow of AMP at the system’s boundaries. This model setup allowed for the investigation of long-term interactions between AMP and the pathogen cell. For both models, simulations resulted in a decrease in AMP uptake by the pathogen cell, showcasing the efficacy of the proposed immune evasion mechanism. Protection of the pathogen against AMP was observed for a wide range of parameter combinations across several orders of magnitude, suggesting that CME acts as a robust immune evasion mechanism.

In order to understand the key processes contributing to CME, we performed a global sensitivity analysis of the model through parameter screening, partial dependence plots, and SHAP analysis. We found two main rate parameters that drive the simulation outcome, namely the AMP uptake rate parameter ${U}_{A}$ and the rate parameter ${S}_{D}$ associated with the secretion of defense molecules, which are both involved in reactions on the pathogen cell membrane. Interestingly, the sensitivity analysis revealed that the association rate parameter ${k}_{{on}}$ between AMP and defense molecule has a relatively low effect on the score ${s}_{\mathrm{AMP}}^{\mathrm{conSDM}}$ within the screened parameter range compared to the other parameters. This result is likely due to the lower bound of the screened value for ${k}_{{on}}$ being already sufficiently high to form complexes thus protecting the pathogen against AMP. The sensitivity analysis revealed the impact of all other parameters on the simulation outcomes: the pathogen benefits from a high secretion of defense molecules, a short delay between AMP uptake and secretion of defense molecules, and a high affinity between defense molecules and AMP. On the contrary, a high uptake rate of AMP and a high dissociation rate of the complexes are unfavorable conditions for the pathogen.

Next, we investigated the role of diffusion in CME mechanism. In particular, we examined the role of the diffusion of complexes to determine whether the passive transport of AMP away from the pathogen cell via complex formation and its diffusion is necessary for pathogen protection, or if the mere formation of complexes is sufficient for immune evasion. To address this, we simulated a scenario where the diffusion of complexes was disabled, ensuring that the complexes remained at their formation site. The simulation of this deliberately unrealistic scenario allowed us to characterize and quantify the role of diffusion of complexes in CME. In this particular case, we found that the diffusion of complexes was beneficial for the pathogen in a majority of the cases, with 70% of the simulations resulting in lower AMP concentration being taken up by the pathogen when complexes were allowed to diffuse compared to prohibited diffusion. This regime was achieved for diffusion-limited simulations, when reactions occur faster than diffusion. As a consequence, complexes predominantly formed close to the pathogen cell surface inducing the transport of AMP within the complex away from the pathogen. For the remaining 30% of the simulations, the diffusion of complexes resulted in higher AMP uptake by the pathogen. This second regime was mainly found when defense molecules were secreted in high amounts or when diffusion occurred faster than reactions, both scenarios resulting in complex formation mainly at a distance from the pathogen.

In the second part of the study, we focused on the case of *C. albicans* infection. The integration of the theoretical model alongside available experiments enabled the in-depth analysis of the AMP evasion by *C. albicans*. The CME models provide mechanistic explanations of what had been observed experimentally. Based on available data, we narrowed down the parameter space of the model to further analyze it. Correlating the probability of survival of *C. albicans*, as given by experiments^21^, with the concentration of LL-37 that was taken up by the pathogen, as provided by simulations, enabled us to compare in vitro and in silico results. Several parameter sets resulted in survival probabilities of the pathogen cell that are in agreement with experimental data, suggesting that *C. albicans* may indeed use CME as a mechanism of defense against AMP. For both LL-37 modeled as a one-time treatment or as secreted by immune cells, CME proved to be an efficient mechanism to protect *C. albicans* from AMP. The comparison of simulations with the two models conCMEval and dynCMEval revealed that CME in *C. albicans* infection was more efficient for a low inflow of AMP during a longer time period as compared to a one-time treatment or a high inflow of AMP for a short time. This result may be partly due to the fact that we assumed unlimited secretion by the *C. albicans* cell in this model, implying that the pathogen secreted Msb2* molecules as long as it sensed AMP. With more experimental data becoming available in future research, it will be interesting to extend the model by including a process on limited secretion of defense molecules. Finally, we investigated the question regarding Msb2* valency for LL-37 binding, and a rigorous comparison to the available experimental data enabled us to estimate that every Msb2* molecule can bind at least eight LL-37 molecules, under the assumption that the binding rate ${k}_{\mathrm{on}}$ between LL-37 and Msb2* does not depend on the size of the complex.

Despite these insights, several parameters in the model for *C. albicans* infection remained unknown. Future experiments could help bridge the gap, such as quantifying the candidacidal efficiency of LL-37 over time. This would allow us to fix the uptake rate ${U}_{\mathrm{LL}37}$, which was identified by the sensitivity analysis as one of the main drivers of immune evasion by the pathogen. This could, for example, be realized via time-resolved survival assays where *C. albicans* cells are incubated with LL-37 in different concentrations and CFU counts measured at various time points.

It is also important to mention that the simulations did not account for the pathogen death during the simulation time. This minimized the importance of the delay of pathogen response upon AMP uptake set by the parameter ${t}^{* }$ in the dynCME simulations. That is because the score ${s}_{\mathrm{AMP}}^{\mathrm{dyn}}$ only accounts for the system at the non-equilibrium steady state and does not depend on the cumulative AMP that is taken up during the whole simulation time. In a real infection scenario, this parameter can still play an important role in the outcome of immune evasion. For example, in the case of extremely high AMP inflow and high uptake by the pathogen, and concomitant extremely long reaction times of the pathogen cell, it may be killed even before starting to defend itself by secreting molecules.

Another important assumption in the context of AMP evasion by CME is that the defense molecules secreted by the pathogen bind to AMP but do not degrade these molecules. This implies that the complexes unbind depending on the dissociation rate for these two molecules. We here showed that molecular degradation is not required to offer protection of the pathogen against AMP. However, if complex formation were to be associated with some AMP degradation, the protective effect for the pathogen would only increase. If experimental insight into this process becomes available in future experiments, this process could be studied and quantified in silico by adding a degradation for AMP to the existing models.

Furthermore, we would like to emphasize that we used a continuous modeling approach based on PDEs to model CME. This modeling approach is particularly well-suited for high concentrations of molecules and, due to their computational efficiency, PDEs make extensive parameter screenings computationally feasible. However, the number of complexes, especially during the early time points of the simulations, can be quite low. Therefore, it could be of interest to investigate CME using an agent-based model, which would offer a more realistic stochastic representation of the system, albeit at the cost of much higher computational resources.

The CME mechanism could be further studied in coinfection scenarios as both species benefit from the protection against the AMP. For instance, *C. albicans* and *E. coli* can co-occur in vulvovaginal candidiasis infections^35^; and in vitro studies have shown that Msb2* offers protection to both pathogen species^21^. This property arises from the fact that *C. albicans* cells produce and release Msb2* molecules in the extracellular space protecting themselves and any other pathogens in their vicinity from LL-37. Furthermore, studying the molecular interactions between host and pathogen could also be applied to other pathogens and infection scenarios, such as *Streptococcus pyogenes*, for which it was shown that it can secrete streptococcal inhibitor of complement molecules that bind to human AMP^36,37^. Taken together, the current molecular-level model could serve as a foundational framework for developing more complex simulations. A multiscale modeling approach, bridging molecular processes with cell population-level behaviors, would represent a natural next step. Such an approach could explicitly simulate AMP secretion by immune cells, spatial distributions of host and pathogen populations, and context-dependent cooperative effects observed during co-infections. This would provide a more comprehensive and realistic representation of host-pathogen dynamics. However, developing such multiscale models involves substantial increases in complexity, computational demands, and careful parameterization. As such, this molecular-level framework establishes a mechanistic basis upon which future multiscale models can be built, offering critical insight into the role of AMP—defense molecule interactions in immune evasion. Finally, the inhibition of defense molecules secreted by the pathogen could be a target for therapeutic interventions and this may be implemented and tested in silico by extending the PDE models presented here.

## Methods

### Model systems

To simulate CME, we consider a model system for a single pathogen cell that is represented as a sphere. While this cell is kept at a fixed position in the center of a three-dimensional cubic environment, molecules diffuse in this spatial environment under periodic boundary conditions and under reflecting boundary conditions at the surface of the pathogen cell.

In what follows, we will first introduce the basic CME model and then present two different scenarios that differ in the initial conditions of the AMP distribution. In the first scenario, the initial condition of the AMP is set to a concentration constant in space around the pathogen. Thus, AMP are diffusing without any net flow in or out of the system environment and we refer to this scenario as conCME. The second scenario is characterized by the absence of AMP at the initial simulation time and its steady inflow at the system boundaries. This scenario will be referred to as dynCME.

In both scenarios, the pathogen cell secretes defense molecules at its surface with a certain rate, while AMP can be taken up at the surface of the pathogen cell with a certain rate. Defense molecules and AMP form complexes with an association rate constant. This reaction is reversible and complexes can unbind depending on the complex dissociation rate constant. This hypothesis that defense molecules do not degrade AMP is supported by experiments that show no substantial reduction in LL-37 amount and no degradation products after 1 h and a half incubation together with Msb2*^21,22^.

### CME: complex-mediated evasion model

PDEs have emerged as a powerful tool for modeling molecule concentrations undergoing diffusion and reaction processes. The basic CME model includes both diffusion terms and reaction terms, that describe AMP interaction with defense molecules to form complexes. This reaction is reversible, and complexes also dissociate to AMP and defense molecule:

1 $$\frac{\partial [A]}{\partial t}={D}_{A}{\nabla }^{2}[A]+{k}_{off}[C]-{k}_{on}[A][D]-{k}_{deg}[A]$$

2 $$\frac{\partial [D]}{\partial t}={D}_{D}{\nabla }^{2}[D]+{k}_{off}[C]-{k}_{on}[A][D]-{k}_{deg}[D]$$

3 $$\frac{\partial [C]}{\partial t}={D}_{C}{\nabla }^{2}[C]+{k}_{on}[A][D]-{k}_{off}[C]-{k}_{deg}[C]$$

Here, $\left[A\right]$, $\left[D\right]$, and $\left[C\right]$ denote the concentrations of AMP, defense molecules, and complexes, respectively. Each of these molecular concentrations diffuses in the system based on their respective diffusion coefficients. In the first part of the study, we assume AMP and defense molecules to be of the same size and complexes to be twice larger in radius, therefore resulting in the following relation for diffusion constants based on the Einstein-Stokes equation: ${D}_{A}={D}_{D}=2{D}_{C}$. In the analysis of the effect of diffusion on the model outcome (see Supplementary Note 2) and in the application to *C. albicans*, the diffusion coefficients of AMP, defense molecules and complexes were computed based on their respective molecular size. Rate constants ${k}_{\mathrm{on}}$ and ${k}_{\mathrm{off}}$ characterize binding and unbinding of AMP and defense molecules, respectively. Finally, all molecules degrade with a rate constant *k*_deg_ that is assumed to be the same for the different species.

We apply periodic boundary conditions at the surfaces of the cubic environment. This mimics a system containing a single pathogen cell surrounded by numerous other pathogen cells at a density that is determined by the size of the cubic environment, which is set to $30\,{\mathrm{\mu m}}^{3}$. This corresponds to concentrations of pathogens typically used in experiments (${10}^{6}$ cells$/\mathrm{mL}$) such as whole-blood infection assays^12^. The pathogen radius was set to ${r}_{\mathrm{pathogen}}=3.5\,{\upmu m}$, which corresponds to the size of a yeast *C. albicans* cell^38^.

At the surface of the pathogen cell, defense molecules are secreted and AMP is taken up by the cell as expressed by Eqs. (4)-(6):

4 $$\frac{\partial [A]}{\partial x}{|}_{\mathrm{memb}}=\frac{{U}_{A}^{* }\,\cdot \,[A{]}_{\mathrm{memb}}}{{D}_{A}}$$

5 $$\frac{\partial \left[D\right]}{\partial x}{|}_{\mathrm{memb}}=\frac{-{S}_{D}^{* }\,\cdot \,[A{]}_{\mathrm{uptake}}^{t-{t}^{* }}}{{D}_{D}}$$

6 $$\frac{\partial \left[C\right]}{\partial x}{|}_{\mathrm{memb}}\,=\,0$$

Here, ${U}_{A}^{* }=\,\sqrt{{D}_{A}\cdot {U}_{A}}$ is the parameter that describes the uptake rate of AMP ${U}_{A}$ by the pathogen cell and is correlated with the damage of the pathogen cell by AMP. ${S}_{D}^{* }=\sqrt{{D}_{D}\cdot {S}_{D}}$ characterizes the secretion rate of defense molecules ${S}_{D}$ by the pathogen cell. The secretion takes place on the pathogen cell membrane from where the soluble molecules diffuse into the extracellular space. The secretion of defense molecules by the pathogen cell is modeled as a function of the uptake of AMP at time ${t-t}^{* }$, $[A{]}_{\mathrm{uptake}}^{t-{t}^{* }}$, where ${t}^{* }$ represents the time needed by the pathogen to start secreting defense molecules after the uptake of AMP, such that for $t < \,{t}^{* }$, no defense molecules are secreted. This delay or lag phase can be as fast as milliseconds if molecules are already pre-formed^39^. We assume that the pathogen cell produces and releases defense molecules if it is under attack, represented by the uptake of AMP. The number of defense molecules secreted is assumed to be proportional to the concentration of AMP taken up by the pathogen cell. If the pathogen cell is not sensing any AMP, it does not secrete defense molecules. We assume that the secretion is also bound by the maximum number of molecules a cell can secrete, which is directly related to the size of the pathogen cell and has been estimated to be of the order $1\times {10}^{5}\frac{\mathrm{\mu mol}}{{\mathrm{sdm}}^{2}}$^40^.

We refer to this CME model as CME with model parameters listed in Supplementary Table 1. It will serve as a basis for the other CMEs introduced in this study.

### conCME: model with constant AMP concentration

This model assumes the initial condition of AMP to be homogeneous in the environment. The initial AMP concentration in the conCME, ${[A]}^{t=0}$, is set to ${10}^{3}{\,{\upmu m}}^{-3}$, corresponding to typical AMP concentrations observed during infection^41^.

### AMP score for conCME simulations

To quantify how well the pathogen can counter the attack by AMP, we compute for the steady state of each conCME simulation the following score: ${s}_{\mathrm{AMP}}^{\mathrm{con}}=\frac{{[A]}_{\mathrm{takenup}}^{t{=}\infty }}{{[A]}^{t=0}}\times 100$ (7). This AMP score evaluates the fraction of AMP taken up by the pathogen cell during the simulation. Since AMP are harmful to the pathogen, we consider this uptake to be directly correlated with the immune-evasion status of the microbe: the lower the concentration of AMP that has been taken up by the pathogen, the higher the pathogen chance of survival. The score is computed at the steady state, which corresponds to the depletion of AMP in the system. This metric allows us to quantify how well the pathogen cell can protect itself from AMP. We can, therefore, compare different scenarios and parameter combinations using the ${s}_{\mathrm{AMP}}^{\mathrm{con}}$ score. This score is a percentage bound from 0 to 100, with low values corresponding to a beneficial regime for the pathogen, and high scores indicating that the pathogen cannot defend itself against AMP. A ${s}_{\mathrm{AMP}}^{\mathrm{con}}$ score of $100 \%$ corresponds to the worst scenario for the pathogen cell, where all the AMP are taken up.

### conCMEval: model for varying binding valency of defense molecules

To incorporate the binding valency of defense molecules, we extended the conCME as follows:

8 $$\frac{\partial [A]}{\partial t}={D}_{A}{\nabla }^{2}[A]+{\sum }_{i=1}^{imax}{k}_{off}[{C}_{i}]-{\sum }_{i=1}^{imax-1}{k}_{on}[A][{C}_{i}]-{k}_{on}[A][D]-{k}_{deg}[A]$$

9 $$\frac{\partial [{C}_{1}]}{\partial t}={D}_{C}{\nabla }^{2}[{C}_{1}]+{k}_{on}[A][D]+\,{k}_{off}[{C}_{2}]-{k}_{off}[{C}_{1}]-{k}_{on}[A][{C}_{1}]$$

10 $$\frac{\partial [{C}_{i}]}{\partial t}={D}_{C}{\nabla }^{2}[{C}_{i}]+{k}_{on}[A][{C}_{i-1}]+{k}_{off}[{C}_{i+1}]-{k}_{off}[{C}_{i}]-{k}_{on}[A][{C}_{i}]$$

11 $$\frac{\partial [{C}_{imax}]}{\partial t}={D}_{C}{\nabla }^{2}[{C}_{imax}]+{k}_{\mathrm{on}}[A][{C}_{imax-1}]-{k}_{\mathrm{off}}[{C}_{imax}]$$

12 $$\frac{\partial [D]}{\partial t}={D}_{D}{{\rm{\nabla }}}^{2}[D]+{k}_{off}[{C}_{1}]-{k}_{on}[A][D]-{k}_{deg}[D\,]$$

Referring to this model as conCMEval, we allow every defense molecule to bind ${imax}$ AMP in one complex. For instance, ${C}_{3}$ is a complex composed of one defense molecule and three AMP. This gives rise to populations in the conCMEval allowing to simulate the impact of the defense molecule binding valency.

### dynCME: model for AMP inflow

In this scenario, we consider a permanent inflow of AMP at the surfaces of the cubic environment. This is realized using Fick’s first law: ${\frac{\partial \left[A\right]}{\partial x}|}_{\mathrm{cube}}=\frac{-{F}_{A}}{{D}_{A}}$, where ${F}_{A}$ denotes the inflow of AMP in the system that are diffusing with constant ${D}_{A}$. The AMP inflow in the dynCME offers a more realistic representation of AMP-secreting immune cells in the vicinity of the pathogen cell as compared to the conCME. The initial concentration of AMP ${[A]}^{t=0}$ is set to $0\,{\mathrm{\mu m}}^{-3}$, with no AMP present in the environment at the start of dynCME simulations. Values for the inflow ${F}_{A}$ are screened over the wide range [10^1^–10^4^] μm^−2 ^· s^−1^, which includes typically observed concentrations of AMP that are secreted by immune cells in the presence of pathogens, in the range[0.2–25] μM^41^.

### AMP score for dynCME simulations

For the dynCME simulations, due to the permanent inflow of AMP at the system boundaries, the previously introduced AMP score ${s}_{\mathrm{AMP}}^{\mathrm{con}}$ cannot be used as defined by Eq. (7), since the initial state is characterized by the absence of AMP in the system. The constant inflow of AMP introduced in the system allows it to ultimately reach a non-equilibrium steady state characterized by a continuous uptake of AMP by the pathogen. We quantify the concentration of AMP taken up by a pathogen per time unit at the non-equilibrium steady state: ${s}_{\mathrm{AMP}}^{\mathrm{dyn}}=\frac{{[A]}_{\mathrm{uptake}}^{t=\infty }}{\triangle t}$, where $\triangle t$ is the time step of the simulation.

This score allows us to quantify how well the pathogen can counteract the inflow of AMP for different parameter regimes. A low value of ${s}_{\mathrm{AMP}}^{\mathrm{dyn}}$ means that the pathogen is taking up only a few AMP per time implying that most of the AMP are neutralized before reaching the pathogen cell, thus showcasing an effective protection against the inflow of AMP. On the contrary, a high ${s}_{\mathrm{AMP}}^{\mathrm{dyn}}$ score indicates that the pathogen cell is constantly taking up a high concentration of AMP and this scenario corresponds to a regime in which the pathogen fails to defend itself against the inflow of AMP. Instead of being effectively transported away in the complex with defense molecules, AMP accumulate in the vicinity of the pathogen cell, ultimately causing its death. All in all, the ${s}_{\mathrm{AMP}}^{\mathrm{dyn}}$ score can be interpreted as a characteristic correlated with the damage done by AMP to the pathogen per time unit, at the non-equilibrium steady state.

### dynCMEval: model with AMP inflow for varying binding valency of defense molecules

Similar to the conCMEval, we introduce the dynCMEval, a model that accounts for the binding valency of the defense molecules. This model allows us to simulate both an inflow of AMP at the system boundary as the dynCME as well as the binding of several AMP by one defense molecule.

In order to compare the results to the conCMEval, the inflow of AMP, regulated by the flux parameter ${F}_{A}$, is switched off after a certain time ${t}_{1}$, which represents the time required for the system to reach a comparable total concentration of AMP to the conCMEval system. In the conCMEval, the initial number of AMP is set to a constant value in space ${{n}_{A}}_{\mathrm{conSDM}}^{t=0}=[{A]}^{t=0}\times {V}_{\mathrm{system}}$, where ${V}_{\mathrm{system}}={c}^{3}-(\frac{4}{3}\times \pi \times {r}_{\mathrm{pathogen}}^{3})$ is the volume of the system. To compute the time needed to reach ${{n}_{A}}_{\mathrm{conSDM}}^{t=0}$ in the dynCMEval based on the inflow ${F}_{A}$, we use the following relation: ${t}_{1}=\frac{\,{{n}_{A}}_{\mathrm{conSDM}}^{t=0}}{6\times {c}^{2}\times {F}_{A}}$, with $6\times {c}^{2}$ the area of the cube boundary. The inflow of AMP is defined as $$\left\{\begin{array}{l}{\frac{\partial \left[A\right]}{\partial x}|}_{\mathrm{cube}}=\,-\frac{{F}_{A}}{{D}_{A}},t\le {t}_{1}\\ {\frac{\partial \left[A\right]}{\partial x}|}_{\mathrm{cube}}=0,t > {t}_{1}\end{array}\right.$$, which allows us to simulate an inflow of AMP until the desired concentration of AMP is reached; then the inflow is stopped for the rest of the simulation.

### Comparison of the probabilities of survival of *C. albicans* in conCMEval and dynCMEval simulations

To compare the distribution of the probability of survival of *C. albicans* between the conCMEval scenario and the different values of inflow of LL-37 of the dynCMEval scenario, a Kruskal-Wallis test was used. The normality of the data was evaluated by the Shapiro test.

## Supplementary information

Supplementary information

Supplementary Movie 1

Supplementary Movie 2

Supplementary Movie 3

Supplementary Movie 4

Supplementary Movie 5

Supplementary Movie 6

## Data Availability

The data from the CME simulations as well as Figures and Supplementary Figures and Videos supporting the conclusions of this manuscript can be accessed here: https://asbdata.hki-jena.de/BachelotEtAl_2025.

## Code availability

The C++ code used for the CME simulations can be accessed here: https://github.com/applied-systems-biology/AMP_evasion_PDE_model.
