# Supplementary material for: Complex-mediated evasion: modeling defense against antimicrobial peptides with application to human-pathogenic fungus Candida albicans
Source: NPJ Syst Biol Appl. 2025 Jul 22;11:81. doi: 10.1038/s41540-025-00559-1 (PMC12284208; doi:10.1038/s41540-025-00559-1)
Supplement: Supplementary file 1 — Supplementary information [file 41540_2025_559_MOESM1_ESM.pdf]

## Supplementary information

# Complex-mediated evasion: modeling defense against antimicrobial peptides with application to human-pathogenic fungus *Candida albicans*

Yann Bachelot[1,2], Anastasia Solomatina[1], Marc Thilo Figge[1,3,*]

[1] Applied Systems Biology, Leibniz Institute for Natural Product Research and Infection Biology, Hans Knöll Institute (HKI), Jena, Germany
[2] Faculty of Biological Sciences, Friedrich Schiller University, Jena, Germany
[3] Institute of Microbiology, Faculty of Biological Sciences, Friedrich-Schiller-University, Jena, Germany

*Corresponding author: thilo.figge@leibniz-hki.de

## Supplementary Movies

**Supplementary Movie 1. Example of a conCME simulation.**
Typical time evolution of the complex-mediated evasion mechanism (CME). The three-dimensional system represents a volume of $27 \times 10^3\ \mu m^3$, with a *Candida albicans* cell of radius $3.5\ \mu m$ centered in the environment. AMP molecules (in blue) are homogeneously distributed around the pathogen at the initial state. The pathogen secretes defense molecules (in red) on its cell surface, and then these molecules diffuse into the extracellular space. Complexes are represented in purple. The total time of the simulation corresponds to a real-time of 2.0 seconds.

The parameter values used to generate this video are described in Table S2. The visualization was made using the software Ovito [1].

**Supplementary Movie 2. Example of a dynCME simulation.**
Typical time evolution of CME. The three-dimensional system represents a volume of $27 \times 10^3\ \mu m^3$, with a *Candida albicans* cell of radius $3.5\ \mu m$ centered in the environment. AMP molecules (in blue) are flowing from the cube sides, towards the pathogen. The pathogen secretes defense molecules (in red) on its cell surface, and then these molecules diffuse into the extracellular space. Complexes are represented in purple. The total time of the simulation corresponds to a real-time of 2.0 seconds.
The parameter values used to generate this video are described in Table S2. The visualization was made using the software Ovito [1].

**Supplementary Movies 3 and 4. Example of conCME simulation for parameter combination where complex diffusion is beneficial for the pathogen**
The videos show the concentration of complex over a simulation time of 0.5 s for a parameter combination which lead to a beneficial regime for the pathogen when complexes diffuse.
Video S2 shows the simulation with complexes diffusing, whereas video S3 shows the simulation without the diffusion of the complex.

**Supplementary Movies 5 and 6. Example of conCME simulation for parameter combination where complex diffusion is prejudicial for the pathogen**
The videos show the concentration of complex over a simulation time of 0.5s for a parameter combination which lead to a prejudicial regime for the pathogen when complexes diffuse.
Video S4 shows the simulation with complexes diffusing, whereas video S5 shows the simulation without the diffusion of the complex.

## Supplementary Figures

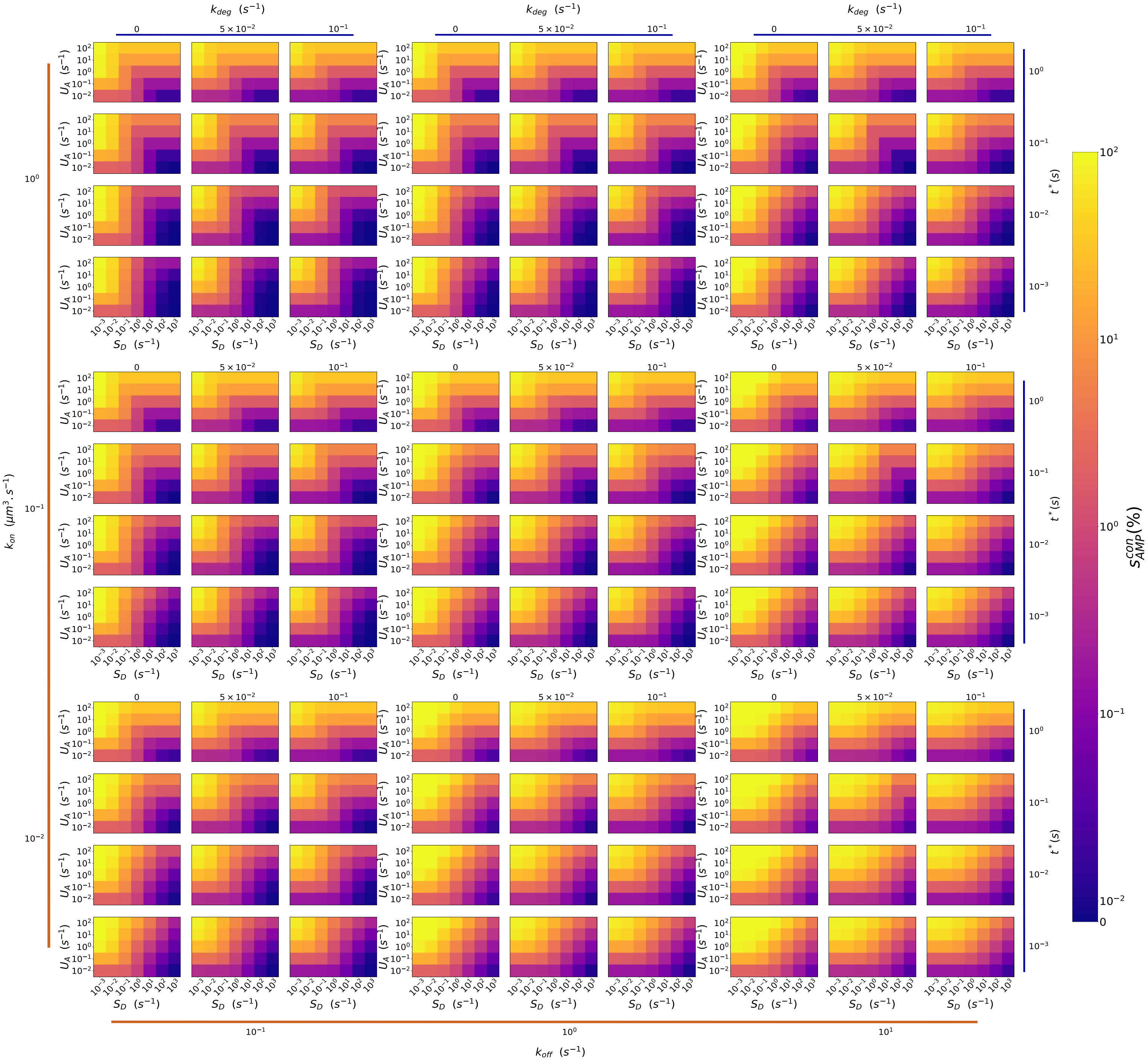


**Supplementary Figure 1. Reaction rate parameter screening of conCME.** Screening over six reaction rate parameters as presented in Supplementary Table 1. The color bar shows the $s_{AMP}^{con}$ score on a log scale.

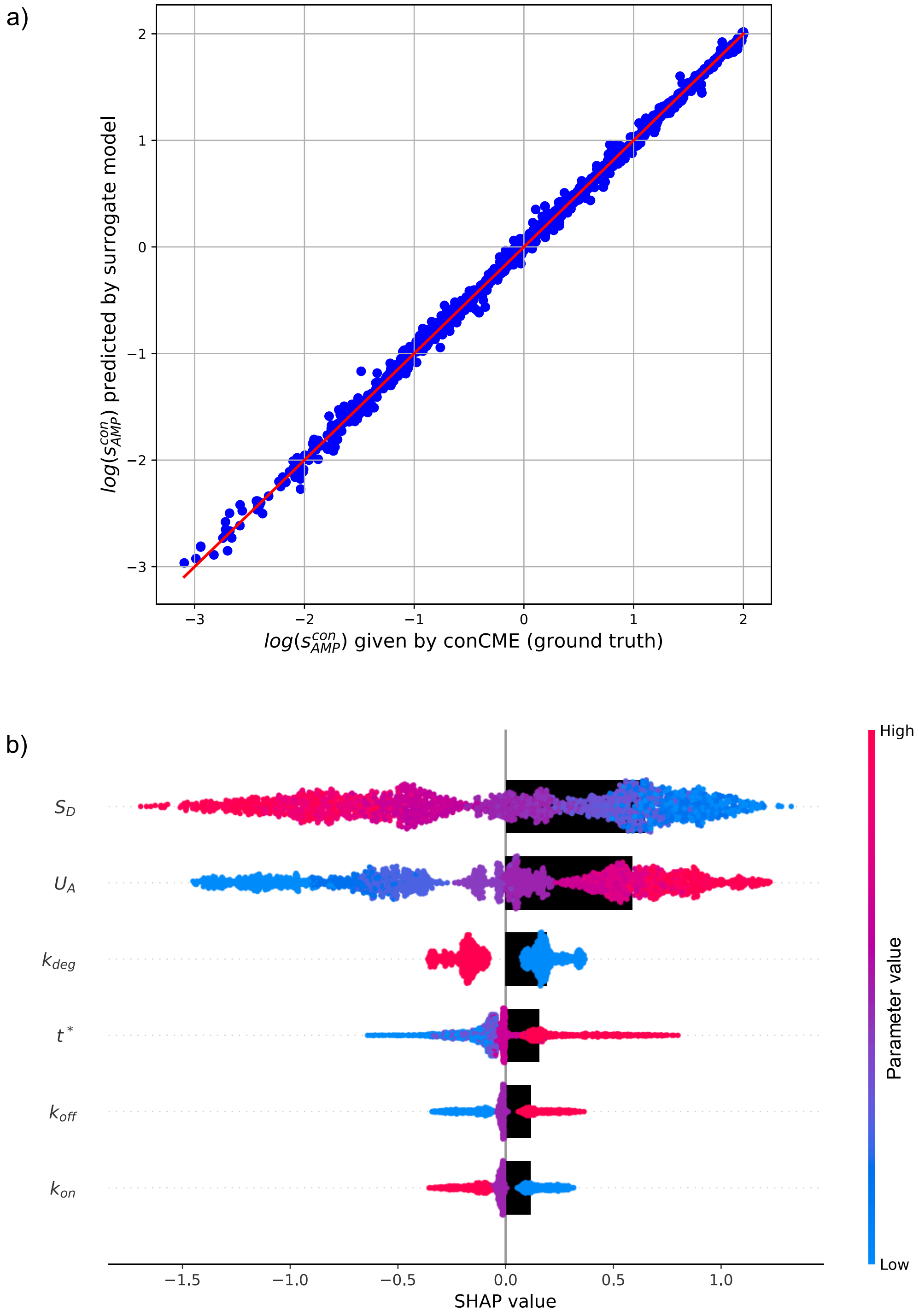


**Supplementary Figure 2. Surrogate model validation and SHAP analysis. a.** Predictions of the $s_{AMP}^{con}$ score by the surrogate model compared to ground truth**.** Each blue point represents a parameter set with the $s_{AMP}^{con}$ score in log scale predicted by the surrogate model on the y-axis and the ground truth $s_{AMP}^{con}$ score in log scale given by the PDE simulation on the x-axis. The red line $y = x$ corresponds to the overlap between the score predicted and the ground truth given by the simulations. **b.** SHAP values analysis of the surrogate model. Black boxes represent the $mean(|SHAP\ value|)$, *i.e.* the mean parameters' contribution importance. Blue and red scatter represent simulations with low and high values for the given parameter, respectively. Negative values indicate a decrease of the $s_{AMP}^{con}$ score, positive values indicate its increase.

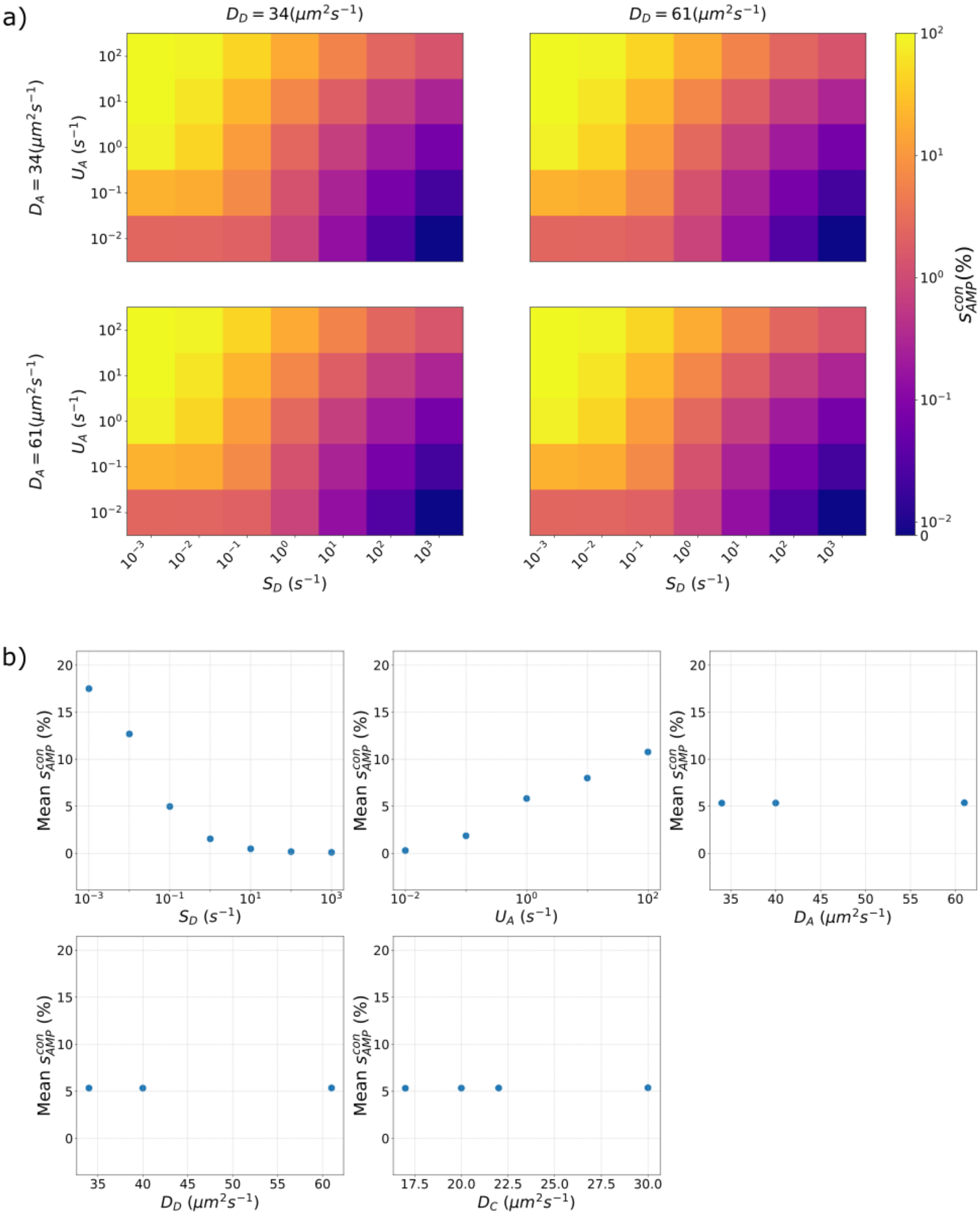


**Supplementary Figure 3. Diffusion coefficients screening of conCME.** Screening over the diffusion coefficients of AMP and defense molecules, as well as reaction parameters $U_A, S_D$. **a.** The color bar shows the $s_{AMP}^{con}$ score. **b.** Partial dependence plots. Each subplot corresponds to one parameter, with the mean score $s_{AMP}^{con}$ (%) computed for each unique parameter value screened.

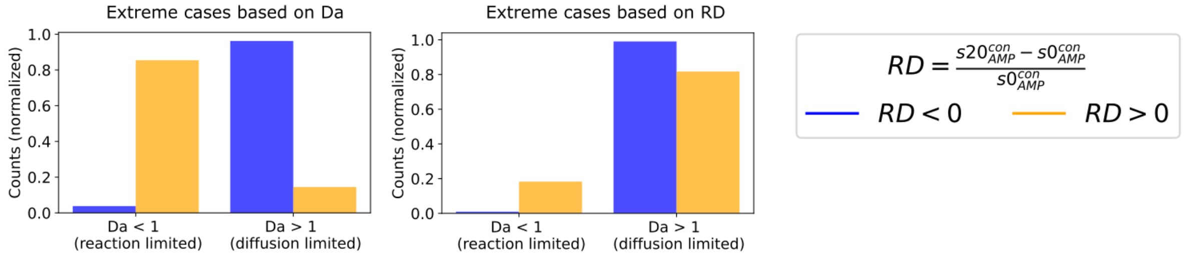


**Supplementary Figure 4. Damkhöler analysis of two regimes: Diffusion of complexes promoting immune evasion ($RD < 0$) or inhibiting immune evasion ($RD > 0$).** The volumes of the parameter space corresponding to the 10% lowest and 10% highest $Da$ simulations (left), and for the 10% lowest and 10% highest $RD$ simulations (right) are shown. For each of the space subsections, color bars show the normalized distributions of the simulations for both $RD$ regimes.

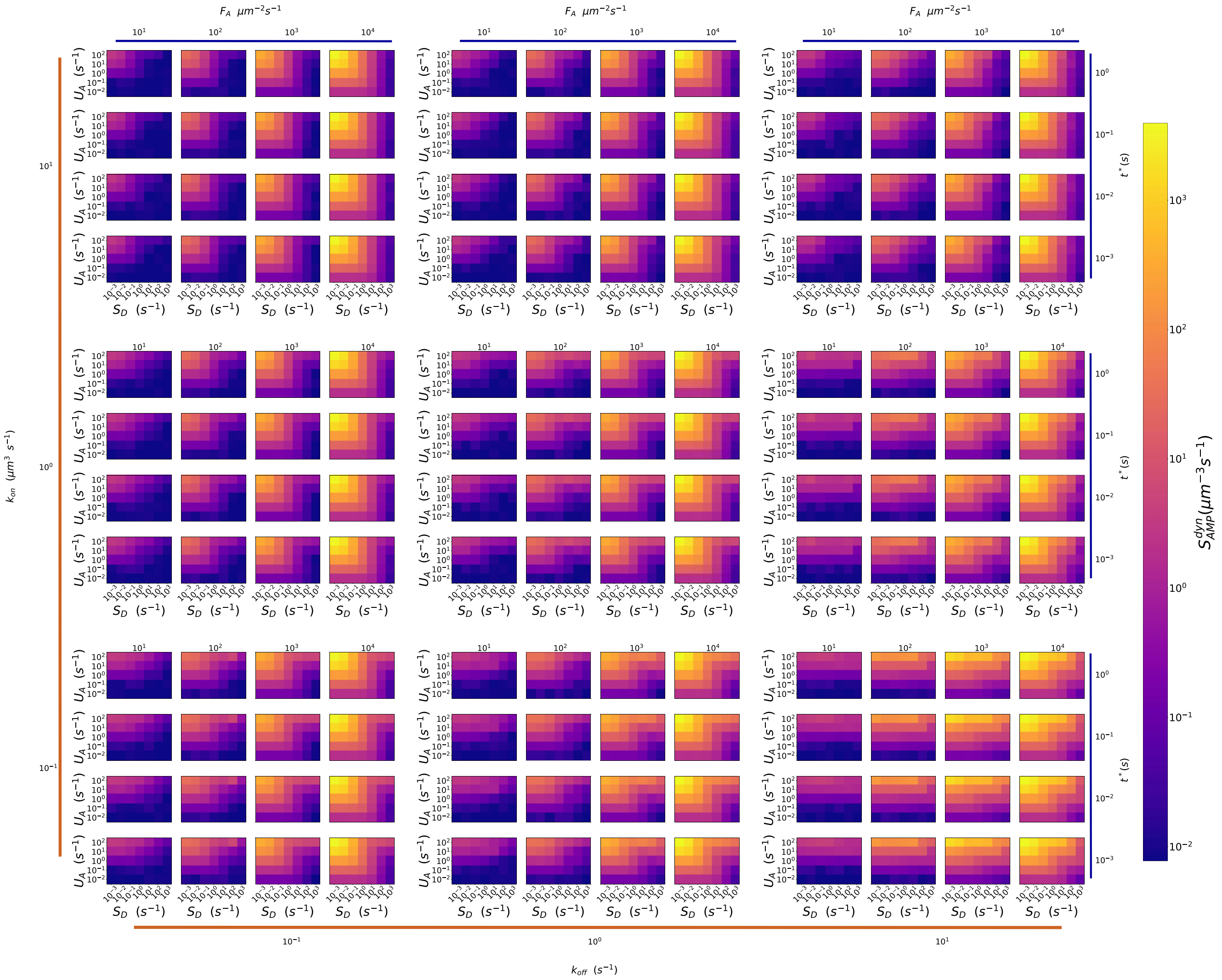


**Supplementary Figure 5. Reaction rate parameter screening of dynCME.** Screening over six reaction rate parameters $U_A, S_D, F_A, t^*, k_{on}$, and $k_{off}$. The color bar shows the uptake of AMP at the steady state on a log10 scale. Low values are shown in dark blue, and high values in yellow.

## Supplementary Tables

### Supplementary Table 1. CME parameters

| Symbol | Description | Range | Unit | Reference |
|---|---|---|---|---|
| $D$ | Diffusion coefficient | 20 – 40 | $\mu m^2 \cdot s^{-1}$ | Stokes – Einstein equation in blood for an average size of 35 amino acids for AMP [2] |
| $k_{on}$ | Association rate constant [AMP – defense molecule] | $10^{-2} - 10^{0}$ | $\mu m^3 \cdot s^{-1}$ | Typical range for protein-protein binding: $10^{6} - 10^{8} M^{-1} s^{-1}$ [3] |
| $k_{off}$ | Dissociation rate constant [AMP – defense molecule] | $10^{-1} - 10^{1}$ | $s^{-1}$ | Typical range for protein-protein dissociation [3] |
| $k_{deg}$ | Degradation rate constant | $0, 5 \times 10^{-2}, 10^{-1}$ | $s^{-1}$ | Extreme values correspond to no degradation and a half-life time of 10 seconds |
| $S_D$ | Parameter related to the secretion rate of defense molecules | $10^{-3} - 10^{3}$ | $s^{-1}$ | Wide range screened |
| $U_A$ | Uptake rate of AMP on pathogen cell membrane | $10^{-2} - 10^{2}$ | $s^{-1}$ | Wide range screened based on [4] |
| $t^*$ | Delay in pathogen's response to AMP uptake | $10^{-3} - 10^{0}$ | $s$ | Wide range screened based on [5] |

### Supplementary Table 2. Parameters value used for exemplary simulation

| *Symbol* | Value |
|---|---|
| $D_A$ | $40\ \mu m^2 \cdot s^{-1}$ |
| $D_D$ | $40\ \mu m^2 \cdot s^{-1}$ |
| $D_C$ | $20\ \mu m^2 \cdot s^{-1}$ |
| $k_{on}$ | $0.1\ \mu m^3 \cdot s^{-1}$ |
| $k_{off}$ | $1.0\ s^{-1}$ |
| $k_{deg}$ | $0.1\ s^{-1}$ |
| $F_A$ | $10^3\ \mu m^{-2} \cdot s^{-1}$ |
| $S_D$ | $10\ s^{-1}$ |
| $U_A$ | $1.0\ s^{-1}$ |
| $t^*$ | $0.25\ s$ |

### Supplementary Table 3. Optimized hyperparameters of the XGBoost model

| Parameter | Range | Optimized value |
|---|---|---|
| *max_depth* | [0, 10] | 9 |
| *learning_rate* | [0.01, 0.5] | 0.0715 |
| *subsample* | [0.3, 1.0] | 0.4634 |

**Supplementary Table 4. Parameters value used for *C. albicans* infection scenario**

| *Symbol* | Value | Reference |
|---|---|---|
| $D_{LL37}$ | $291\ \mu m^2 \cdot s^{-1}$ | Stokes-Einstein equation (s1), in PBS |
| $D_{Msb2*}$ | $91\ \mu m^2 \cdot s^{-1}$ | Stokes-Einstein equation (s1), in PBS |
| $D_{Complex}$ | $69\ \mu m^2 \cdot s^{-1}$ | Stokes-Einstein equation (s1), in PBS |
| $k_{on}$ | $0.1\ \mu m^3 \cdot s^{-1}$ | - |
| $k_{off}$ | $4.4\ s^{-1}$ | Computed from $k_D$ and $k_{on}$ |
| $k_D$ | $44\ \mu m^{-3}$ | Measured experimentally in [6] |
| $S_{Msb2*}$ | $10^{-3} - 10^{3}\ s^{-1}$ | Screened |
| $U_{LL37}$ | $10^{-2} - 10^{2}\ s^{-1}$ | Screened |
| $t^*$ | $10^{-3} - 10^{1}\ s$ | Screened |
| *c* | $30\ \mu m$ | Cube length, computed from the concentration of *C. albicans* in [7] |
| *n* | 9 | Number of LL-37 binding sites on Msb2*, determined in this study |

**Supplementary Table 5. Fitted parameter for the exponential decay function for the relation between the simulated concentration of LL-37 taken up and the experimentally measured probability of survival for *C. albicans* infection for varying uptake parameter**

| $U_{LL37}\ (s^{-1})$ | $k$ fitted |
|---|---|
| $10^{-2}$ | $3.2839 \times 10^{-4}$ |
| $10^{-1}$ | $8.7374 \times 10^{-5}$ |
| $10^{0} - 10^{2}$ | $8.2531 \times 10^{-5}$ |

**Supplementary Table 6. Fitted parameters of the shifted logistic model for the relation between the simulated Msb2* binding valency and the experimentally measured probability of survival for *C. albicans* infection for varying uptake parameter**

| $U_{LL37}\ (s^{-1})$ | $L$ | $k$ | $x_0$ | $c$ |
|---|---|---|---|---|
| $10^{-2}$ | 64.55 | 0.80 | 6.00 | 35.45 |
| $10^{-1}$ | 68.03 | 0.69 | 5.88 | 31.97 |
| $10^{0}$ | 63.45 | 0.60 | 6.51 | 27.22 |
| $10^{1}$ | 12.83 | 0.68 | 8.98 | 26.64 |

## Supplementary Notes

### Supplementary Note 1. Sensitivity analysis based on surrogate model

The conCME was further analyzed by implementing a surrogate model. The idea of a surrogate model is to develop a model that accurately approximates the system behavior in parameter regimes that have not been explicitly simulated. Besides reducing the computation time, the purpose of building a surrogate model is two-fold. First, the surrogate model allows us to predict the outcome of simulations for unseen parameter sets at lower computational costs. Second, a validated surrogate model can be used to study the sensitivity of model predictions on its parameters.

We used an XG-Boost model [8], which is a tree-boosting-based method that has been widely used since its introduction for classification and regression tasks. The hyperparameters such as the depth of the trees and learning rate were tuned using the Optuna framework [9]. The optimized hyperparameters are gathered in Supplementary Table 3. The surrogate machine-learning model was trained on the data presented in Supplementary Figure 1. The supervised learning was done by feeding the model with the parameter sets as input and the $log(s_{AMP}^{con})$ as an output. A 5-fold cross-validation was performed to train and validate the surrogate model. For validation, the results of the model predictions are presented in Supplementary Figure 2a. The mean-squared error (MSE) achieved on the test dataset is $MSE = 1.68 \times 10^{-3}$.

Furthermore, we performed a sensitivity analysis of the surrogate model. The sensitivity analysis was done using SHAP [10], a method based on game theory. The idea behind SHAP is to explain how each model parameter contributes to the model's prediction. This is done by transforming a prediction into the sum of contributions from each parameter. This allows us to gain knowledge of the parameters' importance for the model prediction and to quantify the effect of the conCME parameters on the $s_{AMP}^{con}$ score. The results are presented in Supplementary Figure 2b, with the model parameters (y-axis) ranked by the importance of their contribution regarding the prediction of the simulation outcome. This is indicated by the black boxes in Supplementary Figure 2b, which corresponds to the mean contribution importance of each parameter in the surrogate model predictions. The larger this value, the larger the impact on the prediction of the simulation outcome that the respective parameter has. The importance analysis confirmed that two parameters appeared as the main drivers of the simulation outcome, namely, the uptake rate parameter $U_A$ and the secretion-related rate parameter $S_D$. From the bee-swarm plot in Supplementary Figure 2b, we further characterized how individual parameters affect the $s_{AMP}^{con}$ score. Each dot corresponds to a simulation for a unique parameter set, and for each parameter, the contribution to the model prediction is quantified by a SHAP value plotted on x-axis. The color of each dot relates to the value of the parameter used for individual simulation, whereas its position on the x-axis relates to the effect on the $s_{AMP}^{con}$ score. Note that negative SHAP values indicate a decrease of the $s_{AMP}^{con}$ score, while positive values – its increase.

For the degradation rate parameter $k_{deg}$, two distinct clusters emerged. The blue one on the side of positive values indicates that lower values of the parameter lead to higher $s_{AMP}^{con}$ scores. Similarly, the red cluster in the negative values indicates that higher values of $k_{deg}$ result in lower $s_{AMP}^{con}$ scores. These two separated clusters imply that in every simulation, for the same parameter set, the score is always lower for $k_{deg} = 10^{-1}\ s^{-1}$ than for $k_{deg} = 0\ s^{-1}$. This observation can be explained by the fact that if AMP degrade over time, then they are less likely to harm the pathogen cell. However, since defense molecules and complexes degrade at the same rate as AMP according to the assumption of the conCME, the overall effect of $k_{deg}$ on the $s_{AMP}^{con}$ score was not obvious.

Interestingly, from the bee swarm plot of the parameter related to the secretion rate $S_D$, we can see that all the red dots are clustered on the negative side suggesting high secretion rate parameter values as a strong indicator of low $s_{AMP}^{con}$ scores. However, the blue dots are scattered both on the positive

and negative sides, meaning that low $s_{AMP}^{con}$ scores can also be achieved with low secretion rate parameter values.

**Supplementary Note 2. Quantification of the effect of diffusion**

To analyze and quantify the effect of the diffusion of AMP and defense molecules in CME, we performed simulations by varying these two parameters. We selected the diffusion coefficients based on the size range, in amino acids, of human AMP. Specifically, for the lower bound of this range, which corresponds to 10 amino acids [2], this results in a diffusion coefficient of 61 $\mu m^2 s^{-1}$ in blood, while for the upper bound of 60 amino acids [2], this corresponds to 34 $\mu m^2 s^{-1}$. For complexes, we assumed $r_C = r_A + r_D$, resulting in diffusion coefficients ranging from 17 $\mu m^2 s^{-1}$ to 30 $\mu m^2 s^{-1}$, depending on the combination of sizes used for AMP and defense molecules.

These simulations were then compared to the reference simulations, presented in Supplementary Figure 1, which used diffusion coefficients of $D_A = D_D = 40\mu m^2 \cdot s^{-1}$, corresponding to the diffusion coefficient of an average-sized human AMP of 35 amino acids. We screened over different orders of magnitude for the reaction rate parameters $U_A$ and $S_D$, the two most sensitive parameters in regards to the $s_{AMP}^{con}$ score (see subsection "conCME: Sensitivity analysis reveals reactions on pathogen cell surface driving CME" in the Results section). The other parameters were fixed at the following values: $k_{on} = 0.1\mu m^3 \cdot s^{-1}, k_{off} = 1.0s^{-1}, k_{deg} = 0s^{-1}, t^* = 0.01s$.

The $s_{AMP}^{con}$score was computed for each simulation and represented in Supplementary Figure 3a. The four heatmaps, each one corresponding to a unique combination of diffusion coefficients, show similar $s_{AMP}^{con}$scores for the different values of $U_A$ and $S_D$. This suggests that the diffusion of AMP and defense molecule do not have a large impact on the simulation outcome. We then computed the mean score $s_{AMP}^{con}$ for each unique parameter value, allowing us to assess the overall effect of each parameter on the simulation results using partial dependence plots. The results, shown in Supplementary Figure 3b, indicate that the three parameters $D_A, D_D$ and $D_C$ have close to no effect on the $s_{AMP}^{con}$. The $s_{AMP}^{con}$score of each simulation was also compared to the corresponding reference simulation by computing the relative difference in the $s_{AMP}^{con}$ score $\frac{s_{AMP}^{con} - s_{AMP}^{con}ref}{s_{AMP}^{con}ref}$.

The results show that the maximum deviation observed in the $s_{AMP}^{con}$ lies within 1.2% compared to the reference simulations. This suggests that for molecules within the range of 10-60 amino acids in size, the impact of the diffusion coefficients of the different molecules on the simulations' outcome is minimal.

In conclusion, our screening over diffusion coefficients within the reduced parameter space shows that diffusion has a negligible effect on the simulation results, with deviations in $s_{AMP}^{con}$ remaining below 1.2% for molecules ranging from 10 to 60 amino acids.

**Supplementary Note 3. Damkhöler number analysis**

To further analyze the two regimes found by the analysis of the diffusion of complexes (see subsection "Diffusion of molecular complexes defines two regimes of system behavior" in the Results section), we computed the Damkhöler number $Da$ for extreme cases of the parameter sets, which allows us to compare the system's characteristic diffusion and reaction times. The Damkhöler number is defined as $D_a = \frac{diffusion\ time}{reaction\ time}$. Here the diffusion time is defined as $t_{diffusion} = \frac{l^2}{D}$, with $D$ the diffusion coefficient and $l$ the characteristic length of the system, which in our case corresponds to the distance between the pathogen cell surface and the cube limit.

The reaction time is computed based on the inverse of the highest eigenvalue of the Jacobian matrix $J$ at the steady state: $J = \begin{bmatrix} -k_{on}[D] - k_{deg} & k_{off} & -k_{on}[A] \\ k_{on}[D] & -k_{off} - k_{deg} & k_{on}[A] \\ -k_{on}[D] & k_{off} & -k_{on}[A] - k_{deg} \end{bmatrix}$.

We then have $t_{reaction} = \frac{1}{max(|eigenval(J)|)}$ the characteristic reaction time of the system.

In the left panel of Supplementary Figure 4, the volumes of the parameter space corresponding to the 10% lowest and 10% highest Damköhler numbers ($Da$) were compared. The diffusion-limited regime ($Da > 1$) matches the $RD < 0$ regime, suggesting that the pathogen cell benefits from the diffusion of complexes when the characteristic reaction time of the system is smaller than the characteristic diffusion time. This can be explained by complexes forming prior molecules diffused, *i.e.* close to the pathogen cell surface, creating a gradient of complexes that only then diffuse away. The reaction-limited regime ($Da < 1$) aligns with the $RD > 0$ regime, indicating that when the characteristic time is smaller for diffusion than for reactions, diffusion of complexes becomes disadvantageous for the pathogen cell. In the right panel of Supplementary Figure 4, the Damkhöler number was plotted for the volumes space corresponding to simulations with the 10% lowest and 10% highest $RD$ values. For $RD < 0$, almost all simulations are diffusion-limited. Interestingly, for $RD > 0$, the majority of the simulations are also diffusion-limited, with only a small fraction being reaction-limited. Reaction-limited simulations imply that diffusion of complexes is detrimental to the pathogen cell, while diffusion-limited simulations imply that diffusion of complexes is beneficial to the pathogen cell. These results back up the observations made on the formation of complexes in Figure 5b. Beneficial diffusion of complexes implies diffusion-limited simulations, which was also expected, but disadvantageous diffusion of complexes does not necessarily imply reaction-limited simulations. That is because even though reactions would happen at a faster scale than diffusion, if defense molecules are present in excessive amounts compared to AMP, they would still diffuse and form complexes where AMP are present, *i.e.* far from the pathogen cell.

**Supplementary Note 4. CME parameters derivation**

To estimate the size of the different molecules, we first assume that they have a spherical shape. We can then estimate their radius using their respective molecular weight [11]: $r\,[nm] = 0.066 * M^{\frac{1}{3}}[g/mol]$.

For the first part of the manuscript, we assume a generic human AMP with an average size of 35 amino acids. Based on [12], we assume an average molecular weight of $118.9\,g \cdot mol^{-1}$ per amino acid, resulting in a molecular weight $M_{AMP} = 4.16kDa$. This corresponds to a size of approximately $r_{AMP} = 1.06\,nm$.

With a molecular weight of $M_{LL37} = 4kDa$ , we estimate the radius of LL-37 to be around $r_{LL37} = 1.04\,nm$. For Msb2*, its molecular weight was measured to be around $M_{Msb2*} = 130kDa$, resulting in a radius of $r_{Msb2*} = 3.34\,nm$.

Based on the size of the molecules and considering that the cells are in blood, we can use the Stokes-Einstein equation to obtain their diffusion coefficient:

$D = \frac{k_B T}{6\pi\eta r}$ $(s1)$, where $k_B$ is Boltzmann's constant, $T$ is temperature, η is the system's viscosity and r is the radius of the spherical molecule.

Applying equation (s1) to the generic human AMP in blood, this results in a diffusion coefficient of $D_{AMP} = 40\,\mu m^2 \cdot s^{-1}$.

Applying equation (s1) to LL-37 and Msb2*, we end up with diffusion coefficients in PBS of $D_{LL-37} = 291\ \mu m^2 \cdot s^{-1}$ and $D_{Msb2*} = 91\ \mu m^2 \cdot s^{-1}$, respectively. The different parameters estimated in the case of *C. albicans* in PBS are summarized in Supplementary Table 4.

The size of the 3-dimensional environment was determined by the concentration of *C. albicans* cells used in the experiments performed in [7]. Since only one *C. albicans* cell is considered in the model, we can compute the size of the environment needed to match the experimental setup. The concentration of *C. albicans* cells used for the survival assay is given by optical density $OD600 = 0.3$. This corresponds to approximately $2.4 \times 10^7 cells/mL = 2.4 \times 10^{-5}\ cells/\mu m^3$ present in the solution. This leads to a cube of length $c = \sqrt[3]{\frac{1}{2.4\times10^{-5}}} \approx 30\mu m$ for a single pathogen cell.

## Supplementary References


[1] A. Stukowski, "Visualization and analysis of atomistic simulation data with OVITO–the Open Visualization Tool," *Model. Simul. Mater. Sci. Eng.*, vol. 18, no. 1, p. 015012, Dec. 2009, doi: 10.1088/0965-0393/18/1/015012.

[2] Y. Huan, Q. Kong, H. Mou, and H. Yi, "Antimicrobial Peptides: Classification, Design, Application and Research Progress in Multiple Fields," *Front. Microbiol.*, vol. 11, no. October, pp. 1–21, 2020, doi: 10.3389/fmicb.2020.582779.

[3] C. R. Sanders, "Biomolecular Ligand-Receptor Binding Studies: Theory, Practice, and Analysis," *Dept. Biochem. Vanderbilt Univ.*, pp. 1–43, 2010.

[4] C. J. Richards, T. C. Q. Burgers, R. Vlijm, W. H. Roos, and C. Åberg, "Rapid Internalization of Nanoparticles by Human Cells at the Single Particle Level," vol. 17, 2023, doi: 10.1021/acsnano.3c01124.

[5] M. Shamir, Y. Bar-On, R. Phillips, and R. Milo, "SnapShot: Timescales in Cell Biology," *Cell*, vol. 164, no. 6, pp. 1302-1302.e1, Mar. 2016, doi: 10.1016/J.CELL.2016.02.058.

[6] M. Swidergall, A. M. Ernst, and J. F. Ernst, "Candida albicans mucin Msb2 is a broad-range protectant against antimicrobial peptides," *Antimicrob. Agents Chemother.*, vol. 57, no. 8, pp. 3917–3922, Aug. 2013, doi: 10.1128/AAC.00862-13/ASSET/1D330F4A-D7D1-4997-92A3-7959DD3BEA9B/ASSETS/GRAPHIC/ZAC9991020540003.JPEG.

[7] E. Szafranski-Schneider *et al.*, "Msb2 shedding protects Candida albicans against antimicrobial peptides," *PLoS Pathog.*, vol. 8, no. 2, 2012, doi: 10.1371/journal.ppat.1002501.

[8] T. Chen and C. Guestrin, "XGBoost: A Scalable Tree Boosting System", doi: 10.1145/2939672.2939785.

[9] T. Akiba, S. Sano, T. Yanase, T. Ohta, and M. Koyama, "Optuna: A Next-generation Hyperparameter Optimization Framework," 2019.

[10] S. M. Lundberg, P. G. Allen, and S.-I. Lee, "A Unified Approach to Interpreting Model Predictions".

[11] H. P. Erickson, "Size and shape of protein molecules at the nanometer level determined by sedimentation, gel filtration, and electron microscopy," *Biol. Proced. Online*, vol. 11, no. 1, pp. 32–51, 2009, doi: 10.1007/s12575-009-9008-x.

[12] T. Hachiya, I. Terashima, and K. Noguchi, "Increase in respiratory cost at high growth

temperature is attributed to high protein turnover cost in Petunia x hybrida petals," *Plant, Cell Environ.*, vol. 30, no. 10, pp. 1269–1283, 2007, doi: 10.1111/j.1365-3040.2007.01701.x.
